# Antibodies specific to SARS-CoV-2 proteins N, S and E in COVID-19 patients in the normal population and in historical samples

**DOI:** 10.1099/jgv.0.001692

**Published:** 2021-11-24

**Authors:** Aleksander Szymczak, Natalia Jędruchniewicz, Alessandro Torelli, Agata Kaczmarzyk-Radka, Rosa Coluccio, Marlena Kłak, Andrzej Konieczny, Stanisław Ferenc, Wojciech Witkiewicz, Emanuele Montomoli, Paulina Miernikiewicz, Remigiusz Bąchor, Krystyna Dąbrowska

**Affiliations:** ^1^​ Hirszfeld Institute of Immunology and Experimental Therapy, Wrocław, Poland; ^2^​ Regional Specialist Hospital in Wrocław, Research and Development Center, Wrocław, Poland; ^3^​ VisMederi Srl, Siena, Italy; ^4^​ Department of Nephrology and Transplantation Medicine, Wroclaw Medical University, Wroclaw, Poland; ^5^​ Department of Molecular and Developmental Medicine, University of Siena, Siena, Italy; ^6^​ Faculty of Chemistry, University of Wroclaw, Wroclaw, Poland

**Keywords:** acquired immunity, antibodies, coronavirus, immunology, SARS-CoV-2

## Abstract

Severe acute respiratory syndrome coronavirus 2 (SARS-CoV-2) has spread globally; recognition of immune responses to this virus will be crucial for coronavirus disease 2019 (COVID-19) control, prevention and treatment. We comprehensively analysed IgG and IgA antibody responses to the SARS-CoV-2 nucleocapsid protein (N), spike protein domain 1 (S1) and envelope protein (E) in: SARS-CoV-2-infected patient, healthy, historical and pre-epidemic samples, including patients’ medical, epidemiological and diagnostic data, virus-neutralizing capability and kinetics. N-specific IgG and IgA are the most reliable diagnostic targets for infection. Serum IgG levels correlate to IgA levels. Half a year after infection, anti-N and anti-S1 IgG decreased, but sera preserved virus-inhibitory potency; thus, testing for IgG may underestimate the protective potential of antibodies. Historical and pre-epidemic sera did not inhibit SARS-CoV-2, thus its circulation before the pandemic and a protective role from antibodies pre-induced by other coronaviruses cannot be confirmed by this study

## Introduction

Severe acute respiratory syndrome coronavirus 2 (SARS-CoV-2) has spread globally, affecting millions of people. Its global dissemination in humans, together with its additional potential to infect domestic and wild animals [1, 2], and the ability of coronaviruses to accumulate molecular changes [3, 4], make our hopes for full eradication of coronavirus disease 2019 (COVID-19) difficult to achieve. In this situation, good understanding of the immunology of COVID-19 will be crucial to protect societies through disease prevention and efficient treatment strategies.

SARS-CoV-2, like many other viruses, may either induce a repertoire of symptoms called COVID-19 or propagate asymptomatically, but with transmission to other individuals. Asymptomatic infections can probably occur in a significant but yet unknown fraction of individuals, estimated at 40–45 % [5]. In the majority of patients, infection results in the development of neutralizing antiviral T cell and antibody production, including IgM, IgA and IgG. The efficacy of antibody production in general may correlate to the intensity of disease symptoms [6, 7]. Virus-specific antibodies are considered valid and most relevant to extrapolate virus spread in populations and to anticipate population immunity against COVID-19 [8]. So far, the overall characteristics of the immune reaction to SARS-CoV-2 seem to involve early antibody response with IgM, followed by IgA and IgG. The incubation period, which is characteristic for this virus, does not exclude effective induction of antibodies. Typically, IgM appear between day 8 and 12 after infection, and decrease by week 12, while high IgG levels start around day 14 and last longer [6–8]. Interestingly, this is IgA as the secretory fraction that provides protection on mucosal surfaces, including oropharyngeal cavity, and typical sites of SARS-CoV-2 entry, thus efficiency of IgA production can be one of the determinants of effective and protective response against SARS-CoV-2 [9].

**Fig. 1.**
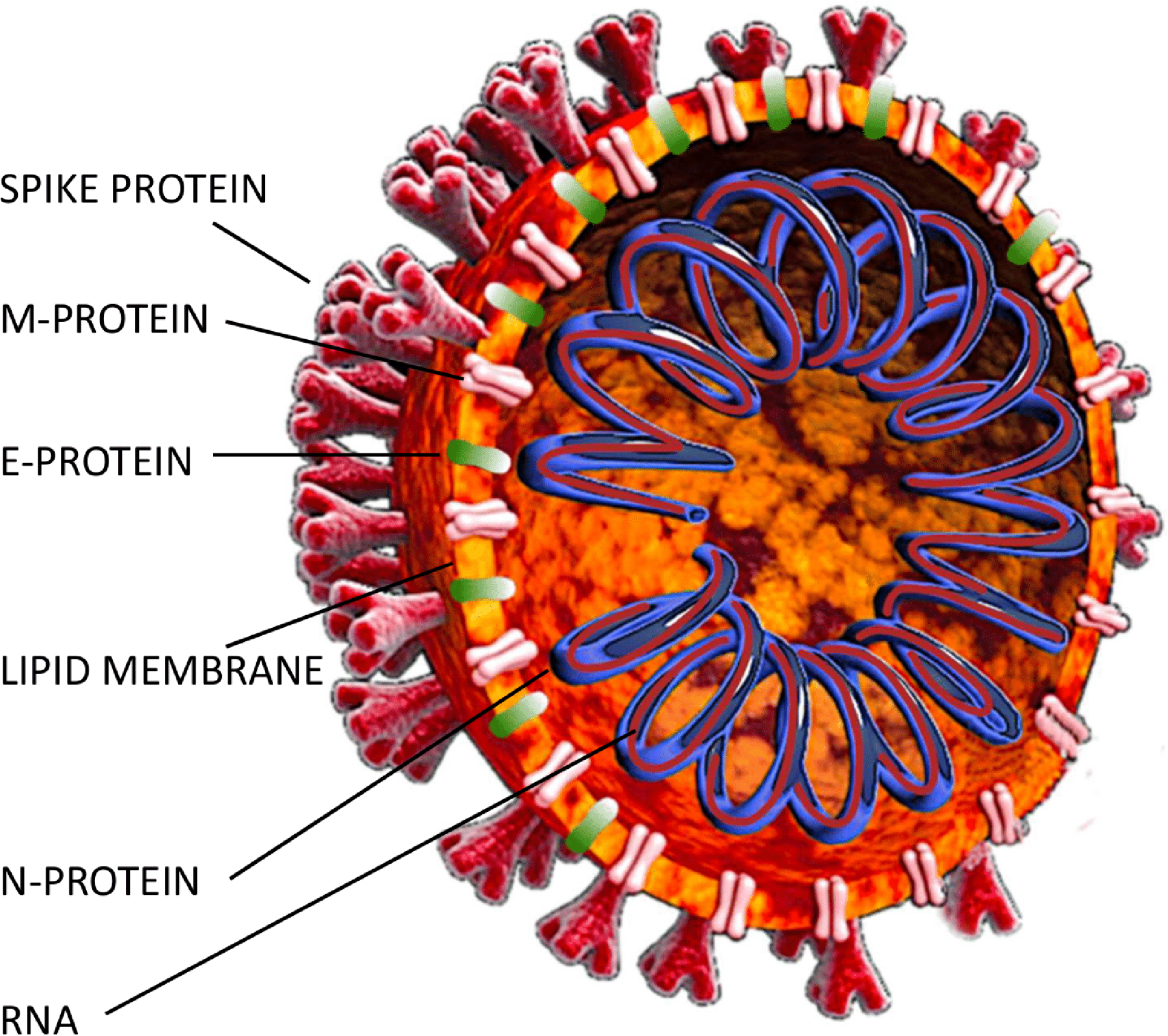
SARS-CoV-2 structural proteins.

Virus-specific antibodies are, however, in fact a pool of antibodies specific to different structural proteins of the virus. The SARS-CpoV-2 virion contains 4 structural proteins: nucleocapsid protein (N), spike protein (S), envelope protein (E) and membrane protein (M) (Fig. 1) [10], and a further 16 nonstructural proteins are also coded by the SARS-CoV-2 genome, although these are less likely to induce an efficient immune response [11]. The common specificity of serological tests for SARS-CoV-2- is the N-specificity, i.e. targeting the N protein that forms a capsid outside the RNA genome. This is mainly due to its high immunogenicity and to the fact that the N protein has been found to be relatively conserved [9, 12], while conserved proteins are obviously good diagnostic targets. The second major target for the immune response is the S protein, with its two major domains: S1 and S2. The S1 subunit function is binding to the receptor on the host cell; it contains the N-terminal domain (NTD) and the receptor-binding domain (RBD). The S2 subunit mediates fusion of the viral and host cell membranes; it consists of fusion peptide (FP), heptad repeat 1 (HR1), central helix (CH), connector domain (CD), heptad repeat 2 (HR2), transmembrane domain (TM) and cytoplasmic tail (CT). Further, the S1/S2 cleavage site plays the key role in virus entry to a host cell. Linear epitopes on the S protein have been demonstrated to elicit neutralizing antibodies in COVID-19 patients [9, 11, 13, 14]. It is protein S with its S1 subunit that binds to human cell ACE2 receptor and thus S-specific antibodies are thought to have the potential to neutralize the virus [15]. Further, antibodies targeting the S1/S2 cleavage site have the potential to impede effective proteolytic activation of the spike protein. It is, however, unclear how other antibody fraction(s) can contribute to neutralization of the virus. For instance, N protein has also been postulated to be applicable for the potential development of vaccines. Here, however, the strategy is not to elicit neutralizing antibodies, but to induce the generation of cytotoxic T lymphocytes capable of destroying infected cells. N-specific antibodies can play a role as effective exposition and response markers rather than effectors. Of note, detection of ‘virus-specific’ antibodies may appear a week before the true capability for virus neutralization is observed [12, 15, 16]. Structural proteins of the most similar virus of the same group, SARS-CoV, induced neutralizing antibodies specific to the protein S only, not those specific to N [17, 18]. On the other hand, the virus-neutralizing potential of sera from SARS-CoV-2 infected patients correlated with N-specific IgG levels in those sera [19]. Further, cross-reactions or cross-neutralization of SARS-CoV-2 by antibodies induced by other coronaviruses (or even other groups of viruses) have been postulated with the hope of limiting the spread of COVID-19 in populations previously affected by other coronaviruses [20–23], but are still unverified.

In this work we seek to understand how protein-specific immune responses to SARS-CoV-2 may contribute to accurate diagnostics, informative epidemiological studies and the real protection of patients. Tested samples were from infected patients (*n*=30, plus subsequent sampling of selected individuals in time), from volunteers representing the normal population (undiagnosed, considered healthy) (*n*=226), and from healthy individuals collected in the years 2010–2017 (*n*=64) or in the pre-pandemic period in Poland from November 2019 to February 2020 (*n*=45) (i.e. historical samples). The first confirmed SARS-CoV-2 infection in Poland was on 4 March 2020, with cases exceeding half a million in early November (https://www.worldometers.info/coronavirus/country/poland/). For these groups, we provide a comprehensive characterization of IgG and IgA specific to structural proteins of SARS-CoV-2: nucleocapsid protein (N), spike protein within its receptor-binding domain (S1) and envelope protein (E), as identified by normalized ELISA, and standard serological diagnostics. Selected samples were tested for their ability to neutralize the virus. Possible correlations between symptoms and severity of COVID-19 and antibody production have been analysed. We present available data on kinetics of antibody production in convalescents. We also report selected cases demonstrating immune response development in selected individuals, including people affected by the virus in one household or in a working environment.

## Methods

Human sera (Table S1, available in the online version of the article).

1. COVID – samples from patients with SARS-CoV-2 infection obligatorily confirmed by RT-PCR, sampling included either patients during active infection or those who had recovered before sampling. This group includes both symptomatic and asymptomatic patients (*n*=30, plus subsequent sampling of selected individuals in time).
2. Healthy/normal population – samples from volunteers without any diagnostic confirmation of SARS-CoV-2 infection before sampling (neither by laboratory testing nor assessment of symptoms by a physician), often with negative results from RT-PCR testing. Some individuals in this group might, however, have been affected by SARS-CoV-2 infection without being diagnosed; they were interviewed for possible COVID-19 symptoms, to exclude oligosymptomatic infections. Of note, this group may contain individuals that had been affected by asymptomatic SARS-CoV-2 and remained undiagnosed (*n*=226).
3. Historical – historical serum samples collected from healthy volunteers in the years 2010–2017 and stored in the Biobank of the Regional Specialist Hospital in Wrocław, Research and Development Center (*n*=64)
4. Pre-epidemic – sera collected between November 2019 and February 2020, i.e. before the first officially confirmed SARS-CoV-2 case in Poland, stored in the Biobank of the Regional Specialist Hospital in Wrocław, Research and Development Center (*n*=45).

Serum was collected into tubes (BD SST II Advance), left for 1 h at room temperature (RT) to clot and separated from the clot by centrifugation (10 min, 2000 ***g***, RT), before storage at −20 °C until further use.

### Bioethics statements

Experiments were approved by the local Commission of Bioethics of the Regional Specialist Hospital in Wrocław (approval numbers: KB/nr2/rok2017, KB/02/2020, KB/03/2020).

### Serological diagnostic tests

The EDI Novel Coronavirus COVID-19 IgG ELISA kit (KT-1032) (EDI, Epitope Diagnostics, Inc., San Diego, CA, USA and the COVID-19 IgM/IgG Duo rapid test (SD Biosensor, Gyeonggi-do, Republic of Korea) were employed according to the manufacturers’ instructions. The tests are N protein-specific (these tests detect anti-N antibodies).

### SARS-CoV-2 proteins

SARS-CoV-2 proteins were used as recombinant products. To investigate the nucleocapsid protein (N), GenBank QHD43423, RayBiotech, Peachtree Corners, GA, USA (cat. no. 230–01104) was used. To investigate the spike protein (S), its human cell-binding domain (S1) was used – GenBank QHD43416 Val16, Gln690, RayBiotech, Peachtree Corners, GA, USA (cat. no. 230–01101). To investigate the envelope protein (E), GenBank QHD43418, MyBioSource, Inc., San Diego, CA, USA (no. MBS8309649) was used.

### Specific antibody level measurement by ELISA

A MaxiSorp 96-well plate (Thermo Fisher Scientific) was covered overnight at 4 °C under sterile conditions with protein N, S1, or E [100 µl/well, 5 µg ml^−1^, in phosphate-buffered saline (PBS)], washed five times (N or S1) or seven times (E) with PBS, blocked for 45 min (N or E) or 60 min (S1) with SuperBlock (Thermo Fisher Scientific) and diluted five times with PBS, 300 µl/well, RT. The plate was washed five times with PBS with 0.05 % Tween 20. Serum samples diluted in PBS (1/100, in the case of reads outside of the reference curve range: 1/200 or 1/400) were added 100 µl/well, in duplicate, incubated at 37 °C for 2 h, and washed with PBS with 0.05 % Tween 20 5 times (N) or 7 times (E), or 10 times (S1). Detection antibody was added 100 µl/well (peroxidase-conjugated AffiniPure goat anti-human IgG or IgA, Jackson ImmunoResearch Laboratories). (IgM was not tested due to its temporary nature and the most dynamic changes in time within antibody classes.) The plate was incubated for 1 h (RT) in the dark, and washed with PBS with 0.05 % Tween 20 five times (N) or seven times (S1 or E). TMB Stabilized Chromogen (Thermo Fisher Scientific) substrate was added (50 µl/well) and incubated for 15–30 min, 25 µl/well of 2 N H_2_SO_4_ was added, and absorbance was measured at 450 nm (main reading) and 570 nm (background). ELISA standard curve calibration was completed with SpectroStar Nano Data Analysis software on the SpectroStar Nano reader, according to the method previously described by Miura *et al.* [24, 25]

### ELISA unit measurement and calculation

To normalize data as ELISA units, each ELISA plate contained the reference curve as validated and described by Miura *et al.* [24, 25]. Briefly, the reference curve consisted of 10 points of twofold reference serum dilutions, from 1 : 100 to 1 : 51 200 (each processed in duplicate), two wells with PBS instead of serum samples served as zero points, and two uncoated wells (virus proteins not added, serum not added) but blocked wells served as blanks. These reference wells were processed with the whole plate as described above. Gen5 software was used to normalize and calculate ELISA units. Reference serum was preselected as highly reactive by comparing raw reads. The same serum sample was used as the reference in all assays and all plates during the whole study.

### Virus neutralization assay in cell culture

*In vitro* neutralization tests against SARS-CoV-2 were carried out under biological safety level 3 (BSL3) containment conditions. SARS CoV-2 2019‐2019‐nCoV strain 2019‐nCov/Italy‐INMI1‐wild-type virus was purchased from the European Virus Archive goes Global (EVAg, Spallanzani Institute, Rome). Serum samples were heat-inactivated for 30 min at 56 °C; twofold serial dilutions, starting from 1 : 10 up to 5120, were then mixed with an equal volume of viral solution containing 100 TCID_50_ of SARS‐CoV-2. The serum‐virus mixture was incubated for 1 h at 37 °C in a humidified atmosphere with 5 % CO_2_. After incubation, 100 µl of the mixture at each dilution was added in duplicate to a cell plate containing a semi-confluent Vero E6 (African green monkey kidney cell line) monolayer. The plates were incubated for 72 h at 37 °C in a humidified atmosphere with 5 % CO_2_.

#### Cytopathic effect (CPE) readout

After 72 h of incubation, the plates were inspected using an inverted optical microscope. The highest serum dilution that protected more than 50 % of cells from CPE was taken as the neutralization titre (reciprocal of the dilution). Factor 5 was considered negative.

### Cell-free *in vitro* testing for serum interference with S protein binding to ACE2 receptor

The SARS-CoV-2 Neutralizing Antibody Detection ELISA kit (Cayman Chemicals, Ann Arbor, MI, USA, cat. no 502070) was used to assess inhibition of binding of S protein to ACE2 receptor. This test contains positive and negative controls; however, serum samples previously identified by virus neutralization assay in cell cultures as positive or negative served as an additional control and reference.

### Online data viewer

The interactive online viewer was prepared in R 4.0.2, using Shiny package version 1.5.0. Data analysis was carried out using the following libraries: readr (v.1.3.1), tidyr (v.1.1.0), tidyverse (v.1.3.0), dplyr (v.1.0.0), data.table (v.1.12.8), plotly (v.4.9.2.1). The full source code is available at the github repository: https://bitly/3gNdXOH.

### Statistics

Data were analysed by analysis of variance (ANOVA) and the Kruskal–Wallis test or the Mann–Whitney U test with the Statistica 8.0 software package (www.statsoft.pl). In the case of individuals sampled a few times, only the first sample (A) was used for general analyses to avoid dependent samples in the analyses; multiple sampling was only used to demonstrate kinetics of antibody induction.

## Results

### Isotypes and specificity to viral structural proteins of antibodies targeting SARS-CoV-2 in the normal population and in SARS-CoV-2-infected patients

Antibodies specific to the structural proteins of SARS-CoV-2 – nucleocapsid protein (N), spike protein domain S1 (S1) and envelope protein (E) – were identified in the sera of patients who had been infected with SARS-CoV-2 (*n*=30) and compared to those without confirmed infections, including 226 healthy (undiagnosed) donors that represent the normal population and 64 historical serum samples collected in the years 2010–2017. Of note, the group of undiagnosed donors that in the health care system are recognized as healthy, may contain those affected by SARS-CoV-2 asymptomatically; this constitutes a study limitation that should be considered. In all samples, IgG and IgA specific to proteins N, S1 and E were identified. Here we present selected aspects of data analysis. All collected medical, epidemiological, diagnostics and research data are available for further analyses in a free viewer online – https://bit.ly/3gNdXOH – or in Table S1.

**Fig. 2.**
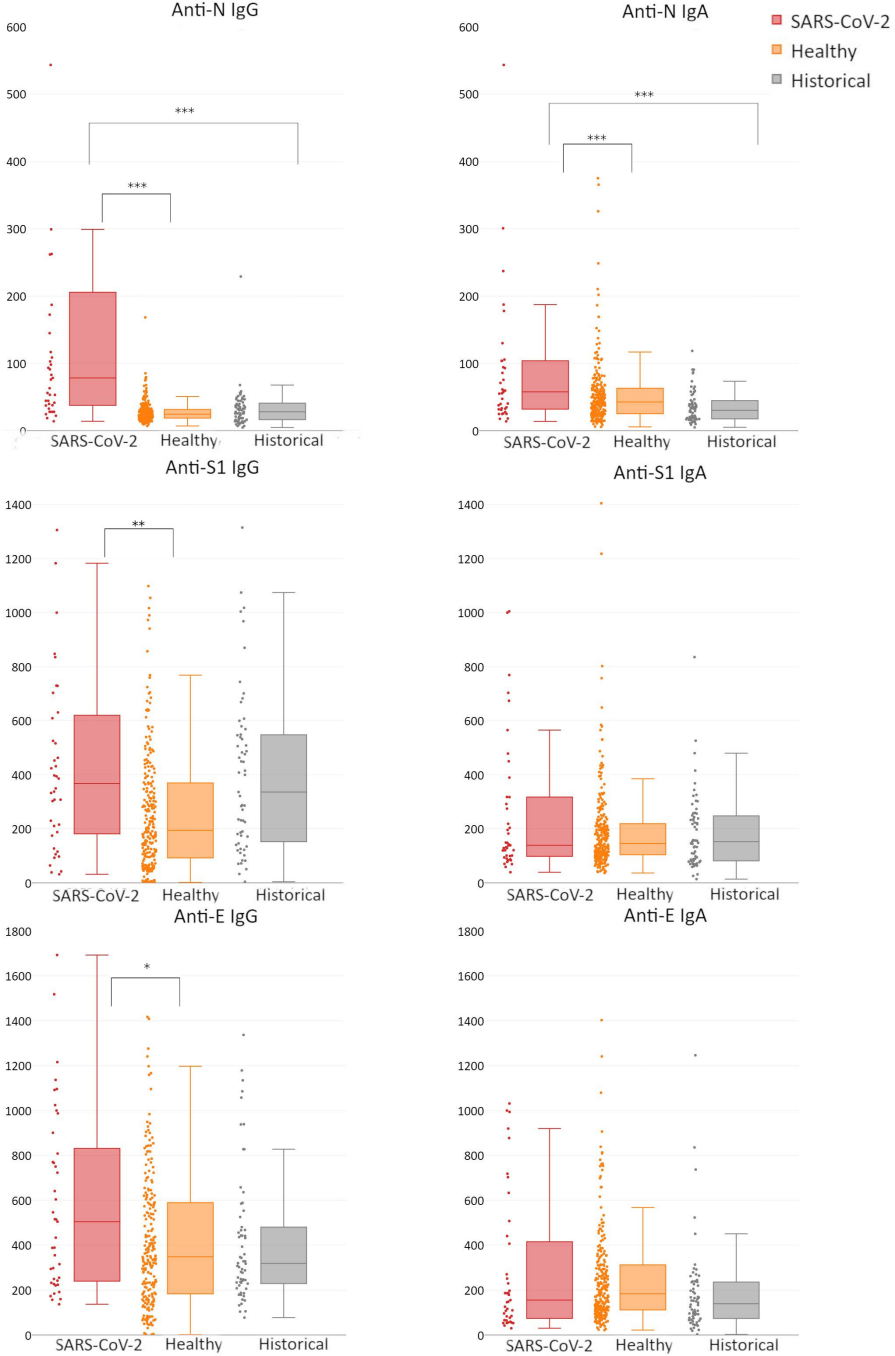
Serum levels of specific antibodies targeting SARS-CoV-2 structural proteins: nucleocapsid protein (**n**), spike protein domain S1 (**s1**) and envelope protein (**e**). Human sera were collected from three groups of patients: SARS-CoV-2, patients after confirmed SARS-CoV-2 infection; Healthy, blood donors without confirmed SARS-CoV-2 infection, blood collected between March and June 2020; Historical, serum samples collected from healthy volunteers in the years 2010–2017. All samples were tested by ELISA on SARS-CoV-2 protein-covered plates, and normalized using reference serum (as described by Miura *et al.* [24, 25]). Normalized ELISA units are presented: vertical lines in the boxes, median; boxes, values within second and third quartile; whiskers, sd; dots at the side of each box, real distribution of individual reads; *, ** and ***, difference between SARS-CoV-2 and Healthy statistically significant in non-parametric ANOVA and Kruskal–Wallis test (*P*<0.05, *P*<0.01 and *P*<0.001, respectively). Specific numerical values of median, mean, quartiles cutoffs, minimal, maximum and N values for each bar are given in Table S2.

We did not find any significant differences in the antibody responses between infected male and female patients (Fig. S1); thus further analyses were conducted without gender grouping. In patients after SARS-CoV-2 infection, both anti-N IgG and anti-N IgA were increased markedly and highly significantly (*P*<0.0000000001 and *P*<0.001, respectively) compared to healthy volunteers (Fig. 2, Table S2). Anti-S1 and anti-E IgG levels in patients after SARS-CoV-2 infection were not markedly but still significantly (*P*<0.01) higher than those in healthy volunteers. Anti-S1 or anti-E IgA did not demonstrate a significant increase in patients infected with SARS-CoV-2 (Fig. 2, Table S2). These observations confirm the high immunogenicity of protein N. Anti-N IgG seems highly applicable for diagnostic testing, while other antibodies seem less reliable or even inapplicable.

**Fig. 3.**
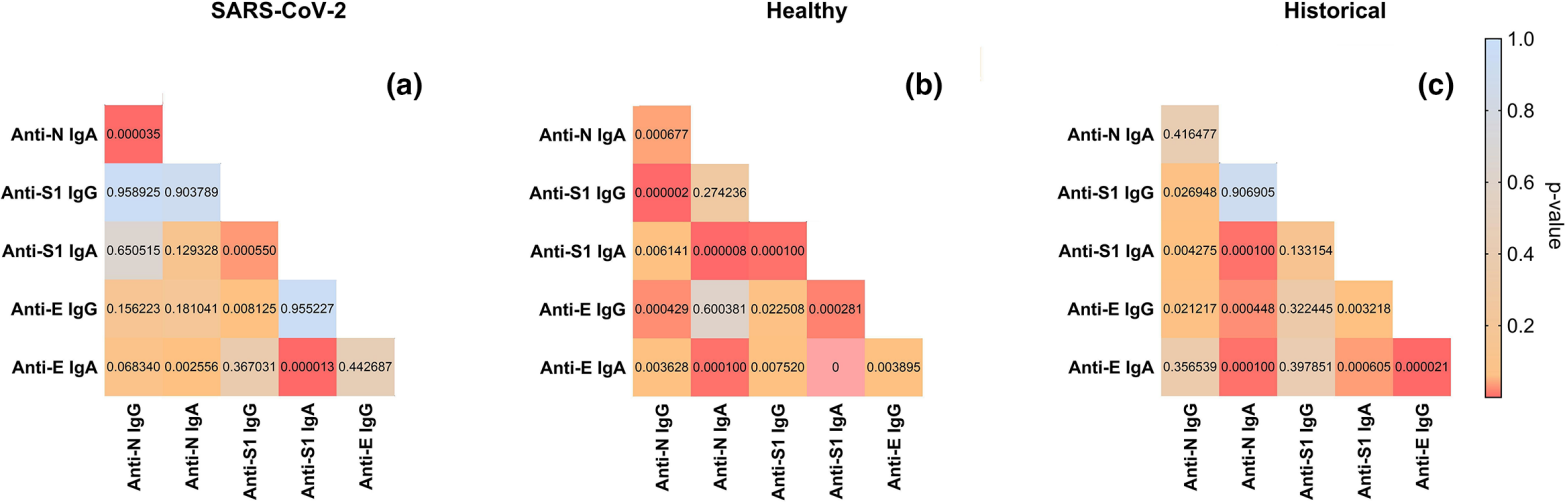
Correlations between serum levels of specific antibodies targeting SARS-CoV-2 structural proteins N, S1 and E in (a) SARS-CoV-2- patients with confirmed SARS-CoV-2 infection; (**b**) Healthy, blood donors without confirmed SARS-CoV-2 infection, blood collected between March and June 2020; (**c**) Historical, serum samples collected from healthy volunteers in the years 2010–2017. Normalized ELISA units are compared, *P*-values calculated by Pearson correlation are presented. Red, strong correlation; blue, weak correlation. Linear regression was used.

Pearson correlation of IgG and IgA levels in all groups is presented in Fig. 3. In SARS-CoV-2-infected patients, correlation of N-specific and S1-specific antibody levels was not observed. This suggests that increase (or fall) of N-specific IgG induced by the infection should not be considered as an indicator of increase (or fall) of the S1-specific fraction of antibodies. Since anti-S1 antibodies are the major fraction predisposed to effective neutralization of the virus, diagnostic detection of anti-N antibodies should not be directly translated to predictions of immunological protection from the virus. On the other hand, in healthy volunteers, the correlation between anti-N and anti-S1 or anti-E IgG was very strong, possibly due to the effect of rare cases of undiagnosed individuals who had, however, undergone infection; they may occur in the investigated population. Historical samples collected within the years 2010–2017 demonstrated some correlations, mostly in the IgA class, but without consistent IgG correlations. This implies that correlations in this group are effects of some unidentified cross-reactions and to some extent random, not a specific reaction (Fig. 3).

Correlations between IgG and IgA of the same specificity were strong in SARS-CoV-2-infected patients with anti-N and anti-S1 antibodies (Fig. 3); IgG represents systemic immunological protection, while IgA may provide mucosal immunity to viral infection and it can also be used in serological diagnostics.

### Medical factors correlating with effective induction of antibodies targeting SARS-CoV-2 structural proteins

**Fig. 4.**
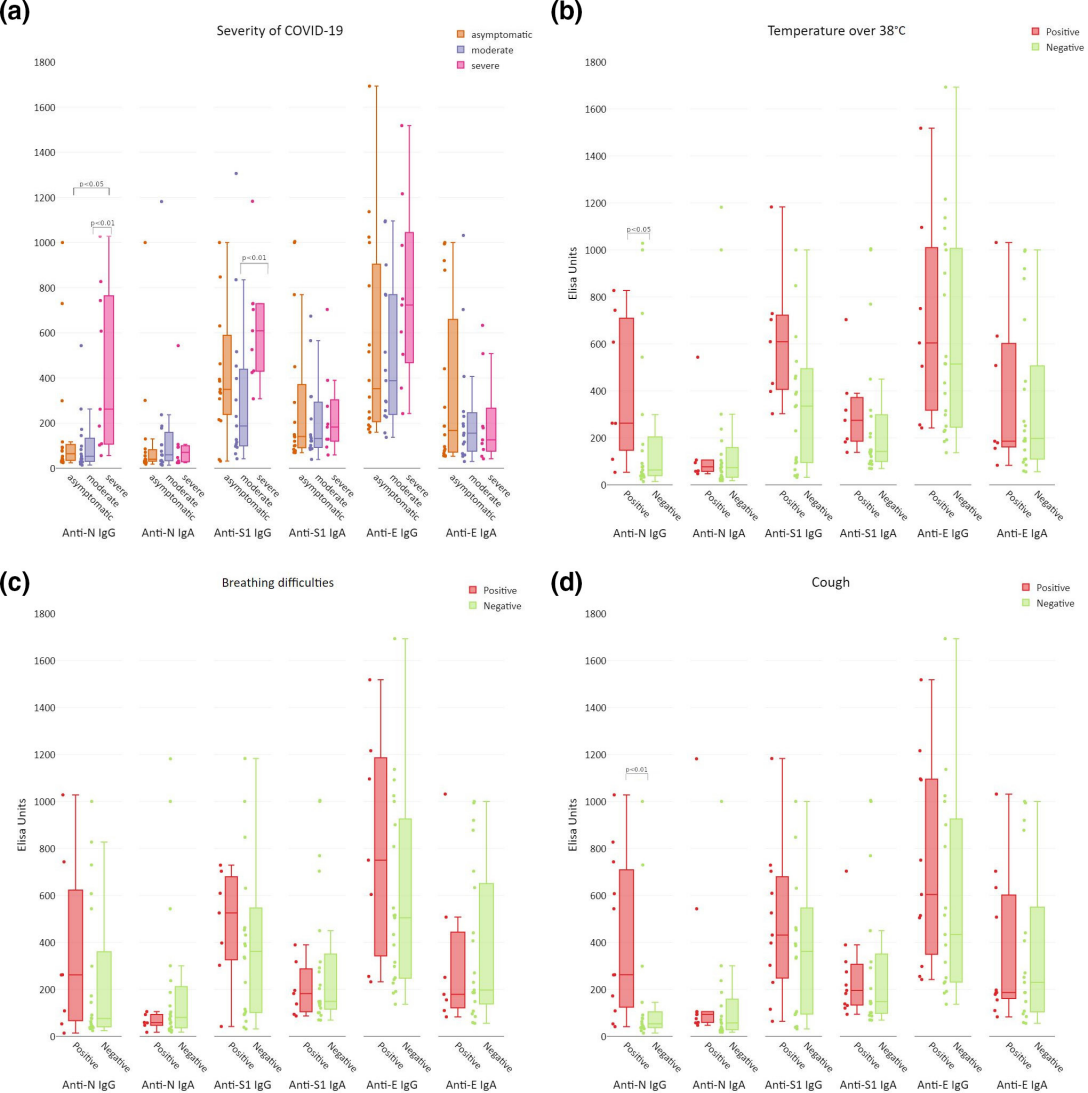
Medical conditions correlating with the induction of antibodies targeting SARS-CoV-2 structural proteins in patients after confirmed infection with SARS-CoV-2. All samples were tested by ELISA on SARS-CoV-2 protein-covered plates, and normalized using reference serum (as described by Miura *et al.* [24, 25]). Normalized ELISA units are presented: vertical lines in the boxes, median; boxes, values within second and third quartile; whiskers, sd; dots at the side of each box, real distribution of individual reads. Statistical analysis by non-parametric ANOVA and Kruskal–Wallis test (**a**) or by Mann–Whitney U test (**b, c, d**). (a) Severity of COVID-19 correlated to increased protein-specific antibody production. Patients were assigned to the groups by the physicians, with regard to symptoms, required treatment procedures, and length of the disease. (b) Rise of body temperature during infection over 38 °C was a predictor of SARS-CoV-2 structural protein-specific antibody induction. Samples from patients after infection were grouped according to observed (positive) or not observed (negative) body temperature over 38 °C. (c) Breathing difficulties during infection was an inadequate predictor of SARS-CoV-2 structural protein-specific antibody induction. Samples from patients after infection were grouped according to observed (positive) or not observed (negative) breathing difficulties. (d) Cough during infection as a positive predictor of SARS-CoV-2 structural protein-specific antibody induction. Samples from patients after infection were grouped according to observed (positive) or not observed (negative) cough in patients.

Overall severity of the disease was assessed and categorized into three groups: asymptomatic, moderate, or severe, according to the supervising physician’s assessment of symptoms and course of the disease (see Table S1). Anti-N IgG was significantly increased in the individuals with severe COVID-19, in comparison to both moderate or asymptomatic cases (*P*<0.01 and *P*<0.02, respectively). Anti-S1 IgG was also increased in the individuals with severe COVID-19, but a significant difference was only observed when compared to moderate manifestation of the disease (*P*<0.002) (Fig. 4a).

Efficient antibody production in patients manifesting particular symptoms was observed. This was primarily in patients with a sharp rise of body temperature (over 38 °C), where a significant (*P*<0.05) increase in N-specific IgG and a non-significant increase (*P*=0.06) in S1-specific IgG was observed (Fig. 4b). Increased antibody induction was not observed in patients who only manifested a body temperature rise over 37 °C but not over 38 °C (*P*=0.3295 and *P*=0.7551 for N- and S1-specific IgG, respectively) (Fig. S2). This strongly suggests that the intensity of the systemic response to the virus manifested by the rise of body temperature can be a predictor of effective antibody response.

Breathing difficulties, which are probably the most dramatic and well-known symptoms of COVID-19, seem, however, to be poor predictors of the development of virus-specific antibodies; their correlation with increased antibody production was not observed (Fig. 4c). In contrast, patients reporting cough had significantly (*P*<0.01) increased levels of anti-N IgG (Fig. 4d). No correlation was observed between antibody production and gastric disorders as a symptom related to COVID-19 (Fig. S3).

### Virus-neutralizing antibodies

Selected sera were tested for their ability to neutralize SARS-CoV-2 in an *in vitro* culture. Sera were blocked (to deactivate the complement system); thus the direct neutralizing effect was studied. Efficient neutralization was only observed in the samples derived from COVID patients (Table 1). Possibilities to select sera highly reactive to only one out of three investigated proteins were limited. In the case of N-specific antibodies, we were not able to select sera with the highest levels of N-specific IgG within the historical collection or healthy volunteers; only COVID samples were tested. They all had virus-neutralizing activity. Within historical samples, those with markedly elevated S1- or E-specific antibodies were found, and they revealed no neutralizing activity against the virus. Further, one COVID patient-derived sample (COV7) with only a high level of anti-E IgG did not neutralize the virus in its culture (Table 1).

**Table 1.** Neutralization of SARS-CoV-2 virus by selected sera in cell culture. High blocking titres indicate neutralizing activity, neutralizing sera highlighted with orange. Sera were selected according to identified levels of SARS-CoV-2 protein-specific IgG specified in the column ‘IgG antibodies status’ (for details see Table S1)

Sample ID	SARS-CoV-2 blocking titre	SARS-CoV-2 infection	IgG antibodies status
COV15	320	**Infection confirmed**	Positive control (all types high)
COV11	640	**Infection confirmed**	Positive control (all types high)
COV18	80	**Infection confirmed**	Positive control (all types high)
HIS178	5	Historical collection of sera (2017)	Negative control (all types low)
371	5	Healthy/undiagnosed	Negative control (all types low)
366	5	Healthy/undiagnosed	Negative control (all types low)
COV3	2560	**Infection confirmed**	Anti-N high
COV24A	80	**Infection confirmed**	Anti-N high
COV22	80	**Infection confirmed**	Anti-N high
HIS114	5	Historical collection of sera (2017)	Anti-S1 high
HIS119	5	Historical collection of sera (2017)	Anti-S1 high
348	5	Healthy/undiagnosed	Anti-S1 high
310	5	Healthy/undiagnosed	Anti-E high
COV7	5	**Infection confirmed**	Anti-E high
262	5	Healthy/undiagnosed	Anti-E high

anti-E high, high level of IgG specific to E protein; anti-N high, high level of IgG specific to N protein; anti-S1 high, high level of IgG specific to S1 protein; negative control, all types of IgG at low levels; Positive control, all types of IgG at high levels.

Identification of virus-neutralizing antibodies was extended with cell-free competitive ELISA (Table S3); the assay allows for detection of antibodies that compete with ACE2 receptor–virus protein S binding. This assay revealed the neutralizing capacity of sera only in patients after confirmed SARS-CoV-2 infection; almost all tested sera were found positive in this group (21 out of 23 tested). Within the historical collection, 12 samples were tested and no neutralizing capacity was detected, in either sera with high anti-N IgG or those with high anti-S1 IgG (Tables S1 and S3) (see also: Case 1, Supplementary File).

These results show that increased levels of S1-reactive or E-reactive antibodies can be found in historical samples, probably due to cross-reactions of antibodies induced by other viruses (or other antigens). However, a protective role of these antibody fractions was not observed herein.

### Kinetics of anti-N and anti-S1 IgG and neutralizing capacity of sera in convalescents

In patients available for subsequent sampling, changes of N and S1 protein-specific antibodies were assessed in time (nine people, time points fitted to the patients’ availability). The approximate moment of infection was derived from the available data (Table S1) and interviews.

**Fig. 5.**
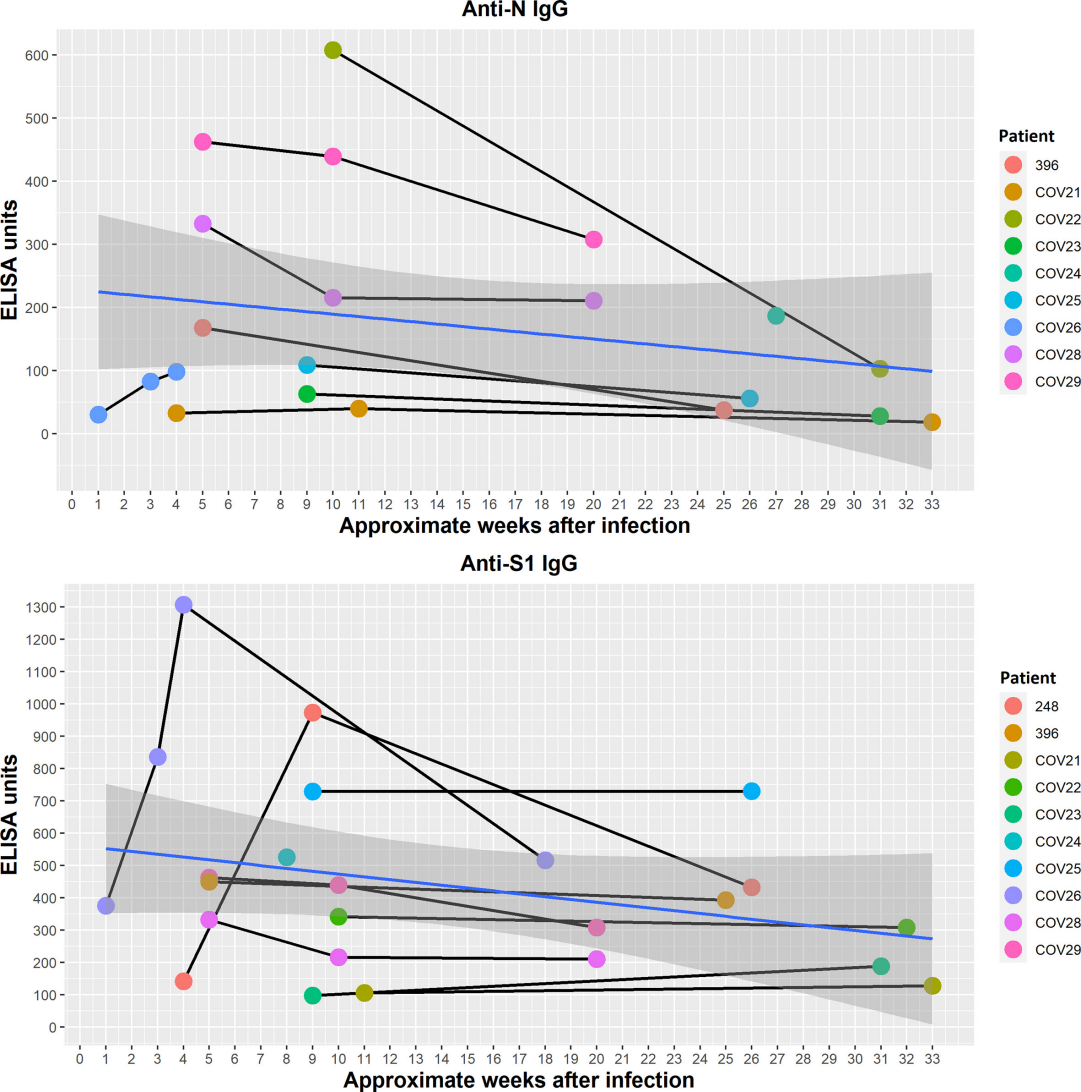
Nucleocapsid (**n**) and spike protein domain (**s1**)-specific IgG antibodies in SARS-CoV-2-infected patients in time. Serum samples were collected from SARS-CoV-2-infected patients available for multiple sampling and time points were fitted to patients’ availability. Sera were tested by ELISA on SARS-CoV-2 protein-covered plates, and normalized using reference serum (as described by Miura *et al.* [24, 25]). Individual values for each patient and time point are presented. Patients are identified by numbers in accordance with Table S1. Time points are presented as approximate weeks after infection. The shaded areas represent the 95 % confidence level interval for predictions from a linear model.

Responses were highly differentiated between patients and the availability of data was limited; however, in most cases IgG levels peaked between 1 and 3 months from the time of infection. Later, levels of N- and S1-specific antibodies tended to decrease (Fig. 5, the shaded areas represent the 95 % confidence level interval for predictions from a linear model), but even then all investigated sera were capable of blocking S protein binding to the ACE2 receptor (Table S3). Thus, the potency to inhibit SARS-CoV-2 infection was still present even more than half a year after infection and in spite of the marked decrease of N-specific antibodies (Fig. 5). This is in line with the fact that the correlation between anti-N and anti-S1 antibody levels was limited. This further demonstrates that changes in N protein-specific IgG have limited value as a predictor of the antiviral potency of patients’ sera.

## Discussion

In the course of SARS-CoV-2 infection, virus-specific antibody induction occurs, as the manifestation of a specific immune response that is the key element of antiviral defences. Virus-specific antibodies are, however, in fact a pool of antibodies specific to different structural proteins of the virus, with different potential as diagnostic targets and as antiviral protection.

In this study we revealed differences in the ability of three SARS-CoV-2 proteins – N, S (S1 domain) and E – to induce specific antibodies, including IgG and IgA isotypes. The major fraction of antibodies induced by the virus in infected patients comprises the N-specific ones, both IgG and IgA (Fig. 2, Table S2). This is in line with observations reported by other groups [17–19]. Antibodies specific to proteins S and E were also significantly increased, but with smaller differences and less significance (*P*<0.01). Thus, this study demonstrates that N-specific antibodies are the best markers of infection, particularly anti-N IgG. These observations are in line with the practice where anti-N IgG are frequently tested in serological diagnostics of COVID-19 [26]. Of note, IgG-based diagnostics require time (days to weeks) for development of the response, while in clinical practice when active infection must be verified, RT-PCR tests should be used. Further, correlations between IgG and IgA of the same specificity have been observed (Fig. 3). Serum IgG represents systemic immunity, while IgA as the secretory antibody provides mucosal protection. Due to technical limitations, secretory IgA testing is not the first choice diagnostics, but it is also possible. Importantly, ELISA-based methods represent a diagnostics tool affected by characteristic limitations. Its reliability decreases with time, when detectable antibody levels naturally decrease in investigated individuals, or when mutated pathogens (variants) with changes in antigenic epitopes emerge and induce antibodies with different specificity.

The key question, however, is how the increased anti-N, anti-S and anti-E antibody levels translate to the potency of virus neutralization. Available reports (including studies on SARS-CoV and SARS-CoV-2) are conflicting, but the majority demonstrate that the S1 domain of the spike protein is the key target for efficient neutralizing antibodies. Correlations of N-specific IgG levels with the virus-neutralizing potential of sera have been demonstrated, but are also assessed as moderate [17–19, 27]. Here, N- and S-specific IgG levels were not correlated (Fig. 3), although N and S proteins are considered to be the two major immunogenic elements of the SARS-CoV-2 virion. The major and strong dependence was the presence of virus-neutralizing antibodies only in patients after confirmed SARS-CoV-2 infection (Table 1 and S3). High reactivity of historical sera to the S1 domain of spike protein, the major viral protein allowing for human cell binding and entry, did not result in virus-neutralizing capacity. Nor did protein E-specific antibodies (Table 1 and S3). Thus, overall high recognition of spike (or envelope) proteins by specific fractions of serum antibodies is not sufficient to inhibit the virus. It seems necessary for a patient to have been exposed specifically to SARS-CoV-2. Probably SARS-CoV-2 infection induces a response to very specific epitopes that play the key role in inhibition of the virus (see also cases 2–4, section: Analysis of selected cases, Supplementary File). This is in line with our observation that only sera from patients with confirmed SARS-CoV-2 infection neutralized (or inhibited) the virus, even when specific IgG levels detected by ELISA were relatively low after a longer time from infection (Fig. 5). Indeed, specific regions of spike proteins that act as the epitopes for neutralizing antibodies have been demonstrated by reaction with COVID-19 patient-derived antibodies; they were mainly located within S1 domain, both within NTD and RBD [9, 28]. Depending on localization within the protein, antibody binding to some regions can be, in contrast, ineffective for neutralization, typically when antibodies bound to these regions cannot efficiently block viral entry to the host cell. N protein has also been postulated as a potential target for vaccination, but the strategy aimed to generate cytotoxic T lymphocytes capable of destroying infected cells. In that case, N-specific antibodies could play a role as effective exposition markers rather than potential effectors [16, 29]

A marked decrease in both anti-N and anti-S1 IgG can be observed within approximately half a year after infection (Fig. 5). However, all late-tested sera revealed virus-inhibitory activity (Table S3). This demonstrates that decrease of N protein-specific IgG with time has limited value as a predictor of the antiviral potency of patients’ sera and it again suggests that only some specific epitopes within protein S are crucial for induction of virus-neutralizing antibodies. A recent report concerning virus neutralization longevity studies showed that neutralization was still observed in almost 43 % of patients 1 year after infection, although the highest titres for neutralizing activity were observed 3 to 4 months after infection [30]. Further, the data indicate that RT-PCR-based testing is most important for diagnosis of active infection, and testing for SARS-CoV-2-specific antibodies is most important for assessment of the degree of protection. In addition to natural loss of neutralizing antibody levels with time, antigenic changes in subsequent virus variants also contribute to decreased capacity of specific antibodies for effective protection against the disease. This should be an important element of future serological investigations of SARS-CoV-2. Analysis of symptoms manifested by COVID-19 patients revealed that anti-N and anti-S IgG induction was correlated with the severity of the disease (other antibody fractions were not) (Fig. 3); this is in line with other reports [30, 31]. In selected symptoms, only a sharp rise of body temperature (over 38 °C) and cough correlated with increased anti-N IgG. Interestingly, breathing difficulties, which are probably the most well-recognized symptoms of COVID-19, did not correlate with increased responses, thus seeming to be a poor predictor of antibody production. Possibly profound dysfunctions in lungs (dyspnoea) are somehow linked to poor protection from the immune system, while cough only, in fact considered to be a less severe symptom, may suggest better immunological protection.

Please note that we have presented selected aspects of data analysis here. All collected information on medical conditions, epidemiological factors and immune response are available for any further analyses in a free viewer online: https://bit.ly/3gNdXOH or in Table S1.

## Supplementary Data

Supplementary material 1
