# Supplementary material for: Antibodies specific to SARS-CoV-2 proteins N, S and E in COVID-19 patients in the normal population and in historical samples
Source: J Gen Virol. 2021 Nov 24;102(11):001692. doi: 10.1099/jgv.0.001692 (PMC8742988; doi:10.1099/jgv.0.001692)
Supplement: Supplementary material 1 [file jgv-102-1692-s001.pdf]

Supplementary material

Analysis of selected cases

Case 1, pre-epidemic patients (patients E181, E196, E198, E201, E222, E226, E227).

Sera collected between November 2019 and February 2020, i.e. shortly before the first officially confirmed SARS-CoV-2 case in Poland that was registered on March the 4th, were tested with the serological diagnostic test (anti-N IgG). In total, 45 serum samples were tested, but only one (E198) was found positive (data not shown). This result could suggest that COVID-19 cases occurred in Poland before the first officially confirmed case. The positive sample E198 and 7 more samples with relatively high reads in the diagnostics were selected for further investigation in this study. However, all anti-S1 IgG levels in these samples were found very low (see: Supplementary Table S1). Sample E198, and in addition samples E182 and E226, were tested by the cell-free inhibitory ELISA, but no virus-neutralizing activity was observed (Table S3). Thus, positive serological diagnostics for SARS-CoV-2 in serum E198 is rather a false-positive or cross-reaction, and it does not demonstrate pre-epidemic circulation of the virus in the population.

Case 2, limited disease transmission in a household (patients 395, 396, COV24, a family in one household).

A 47-year-old woman (patient COV24) developed COVID-19 with a range of symptoms including cough, breathing difficulties, smell and taste loss. SARS-CoV-2 infection was confirmed by RT-PCR testing, no hospitalization required (home isolation and treatment), serological testing when recovered (approx. 1 month from symptoms): positive. Serum demonstrated inhibitory activity against S-ACE2 binding.

Her 46-year-old husband (patient 396) had no symptoms from the respiratory tract and he was confirmed negative by double RT-PCR testing, he was however affected by acute gastric disorders, initially not considered to be linked to COVID-19. Serological testing when recovered: positive; serum demonstrated inhibitory activity against S-ACE2 binding.

Their 21-year-old daughter (patient 395), with no symptoms, confirmed negative by double RT-PCR testing, serological testing: negative. Serum activity against S-ACE2 binding – test inconclusive (tested twice).

In this family transmission from the wife to her husband seems evident, even though SARS-CoV-2 was not detected by his molecular diagnostics. He was affected by a rare type of symptoms and he developed anti-SARS-CoV-2 antibodies, as detected by serological diagnostic tests. SARS-CoV-2 infection in this patient was also revealed by detailed ELISA testing for anti-N, anti-S1, and anti-E antibodies. Particularly, this testing demonstrated that IgG specific to N, S1, and E protein reached 168.2, 450.1, 539.7 [EU], respectively, which is far above median reads in the group of healthy (negative) donors, and even in the group of COVID-confirmed patients (Table S2). Most interestingly, the 21-old daughter, though seemingly not infected and diagnosed as negative by both molecular and serological testing, developed very high levels of IgG specific to proteins S1 and E: 638.8 and >1000 [EU], respectively. On the other hand, however, virus neutralizing activity of the serum was

not confirmed due to inconclusive results (Table S3). These observations suggest that her exposure to the virus resulted in challenging the immune system and in the response, but she was also effectively protected from the disease by other elements of the immune system (no symptoms, negative molecular testing). Weak inhibitory action of induced antibodies may suggest that particularly other (than antibodies) types of immune responses, including cellular response, might play a major role in the protection. Also, non-inhibitory antibodies such as anti-E antibodies may cooperate with non-specific elements of the immune system, including the complement system or phagocytes, and boost their action against the virus.

Case 3, possible asymptomatic infection with immune response to S1 but not to N protein (patient 248 samples A, B, and C)

A 49-year-old healthcare worker (male) exposed to SARS-CoV-2 in the working environment due to a burst of infections in the department. This patient was not affected by any symptoms around the exposure and testing, though he reported some flu-like infection in February (more than 2 months before). Five diagnostic RT-PCR tests within 6 weeks after the exposure were confirmed negative. The first sample for serological testing (A) was collected at the beginning of the infection burst in the department. In this sample N-specific IgM was detected by the diagnostic test, but IgG was not detected either in sample A, or in B (1 month after A) or in C (5 months after A). Indeed, detailed testing of anti-N IgG by normalized ELISA revealed no marked levels (14.0, 25.7, and 28.0 [EU] in samples A, B, and C, respectively), but anti-S1 IgG reached 972.7 [EU] in sample B, which is almost 3 times higher than the median in COVID-confirmed patients (Table S2). Later (sample C) anti-S1 IgG markedly decreased to 432.0 [EU] (56% of loss), being however still higher than the median in COVID-confirmed patients (Table S2). Serum activity against S-ACE2 binding test was inconclusive, i.e. neither positive nor negative (Table S3).

This case is similar to previously described patient 395 (see case 2) and it suggests again that exposure to the virus resulted in an efficient immune response that protected against the disease (no symptoms), but with immunological elements other than antibodies. Neither 'diagnostic' (anti-N) antibodies nor virus inhibitory antibodies were effectively induced, though an overall anti-S IgG rise was evident. This again suggests protective action of the cellular part of the immune system or an indirect inhibitory effect on the virus that can be exerted by non-neutralizing antibodies.

Case 4, repeated infection (patients COV19 and COV20).

Two patients from one household (a husband and a wife) were infected with SARS-CoV-2 in April as confirmed with RT-PCR diagnostics, with a set of typical but rather mild symptoms. After this infection serological diagnostics was negative, and detailed serum testing for SARS-CoV-2 protein-specific antibodies by ELISA revealed relatively low levels of anti-N IgG. Anti-S IgG, however, seemed elevated, reaching 847.6 EU (COV19) and 452.8 EU (COV20). Testing for inhibitory activity against S-ACE2 binding gave inconclusive results. Half a year later (in early November) these two patients were again infected with SARS-CoV-2 as confirmed with RT-PCR; this time diagnostic anti-N testing confirmed N-specific IgG. Surprisingly, significant increase in anti-S1 IgG was not observed (up to 354.5 EU and 400.5 EU in COV19 and in COV20, respectively). However, their sera after the second infection revealed strong inhibitory activity against S-ACE2 binding (Table S3). This observation

advocates that overall levels of S1-specific IgG is not a fully valid predictor of virus-neutralizing potential of patients' sera.

The major question in this case is whether the first infection was not COVID-19, with only a false-positive RT-PCR testing result, or their immune responses during the first infection was impaired, or for the second time they were infected in a mutated and thus different strain of the virus. The latter scenario seems improbable since all testing (on both samples from spring and from autumn) was done with the same proteins and the same specificity was tested but with different results. Since inhibitory activity of sera collected in the spring was in fact not found negative (inconclusive results are considered 'too high for negative, too low for positive'), it seems probable that these particular patients responded to the virus, but they responded insufficiently. The second infection, however, induced a much stronger and effective immune response and virus-specific antibody production.

Supplementary figures

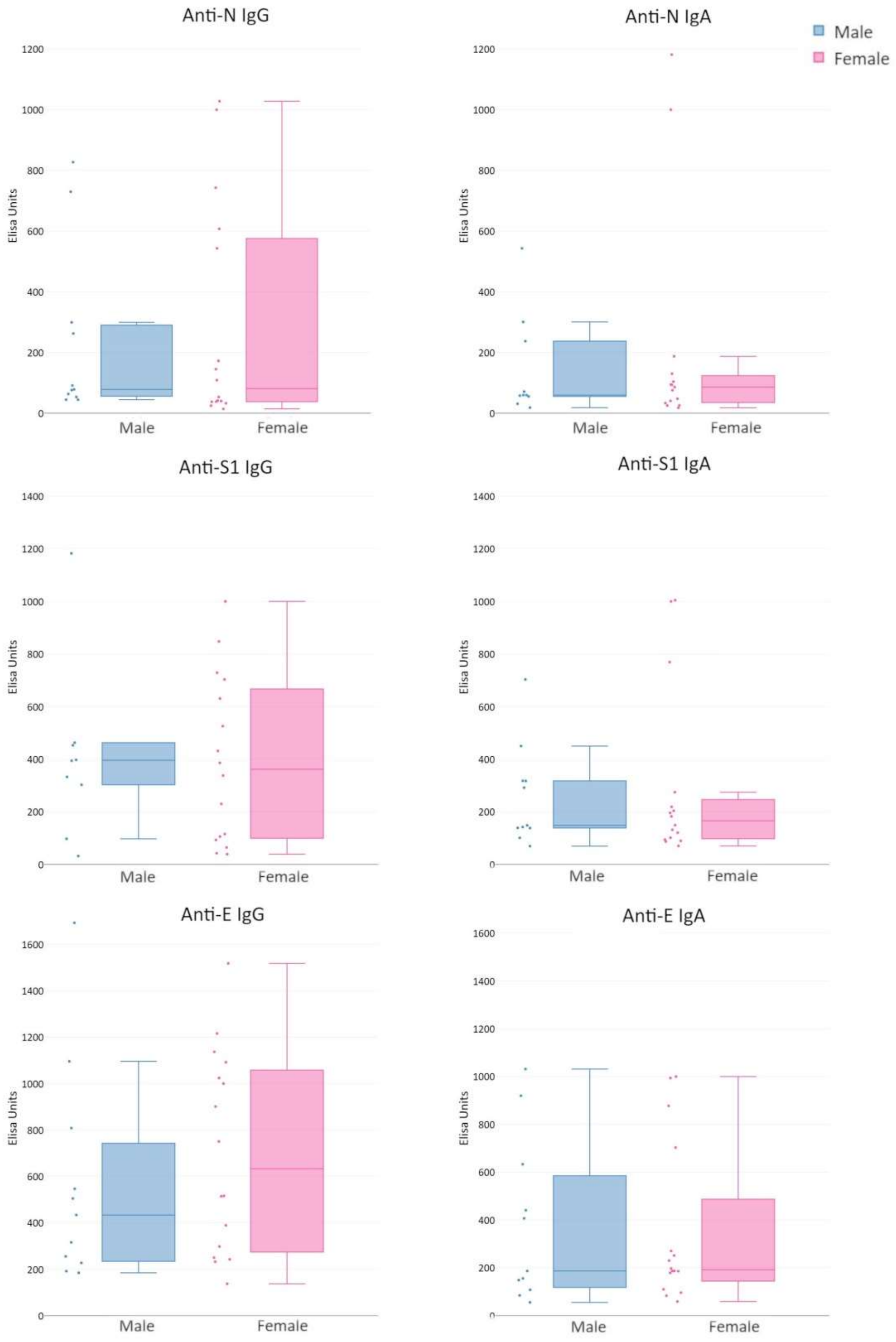

Figure S1. SARS-CoV-2-specific antibody induction in male or female individuals infected with the virus. Statistically significant differences were not found (data in Supplementary Table S1). Normalized ELISA units are presented, vertical lines in the boxes – median, boxes – values within 2nd and 3rd quartile, whiskers – SD, dots at the side of each box – real distribution of individual reads.

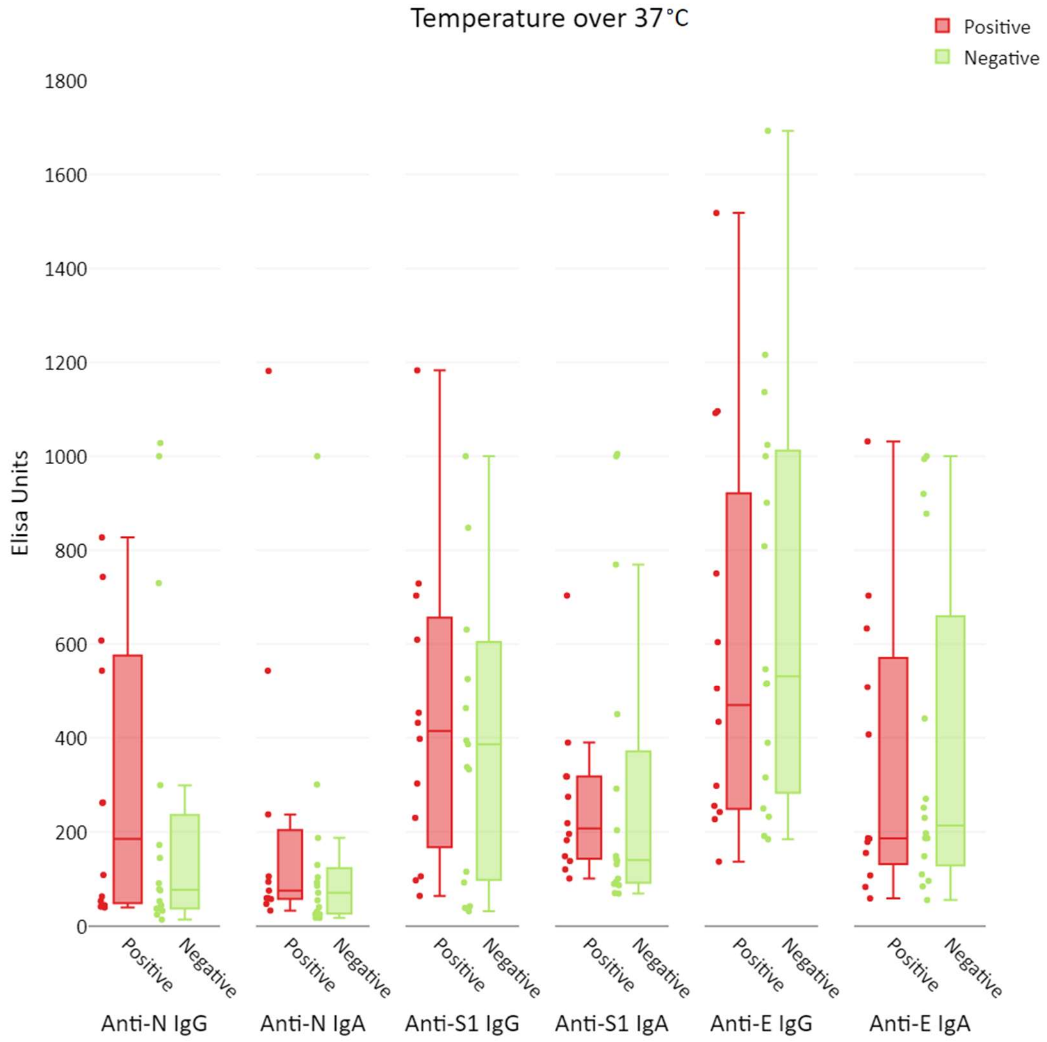

Figure S2. Rise of body temperature during infection over 37°C as a predictor of SARS-CoV-2 structural protein-specific antibody induction. Patients were grouped according to observed (positive) or not observed (negative) body temperature over 37°C. All samples were tested by ELISA on SARS-CoV-2 protein-covered plates, and normalized using reference serum (as described by Miura et al. [1,2]); normalized ELISA units are presented, vertical lines in the boxes – median, boxes – values within 2nd and 3rd quartile, whiskers – SD, dots at the side of each box – real distribution of individual reads.

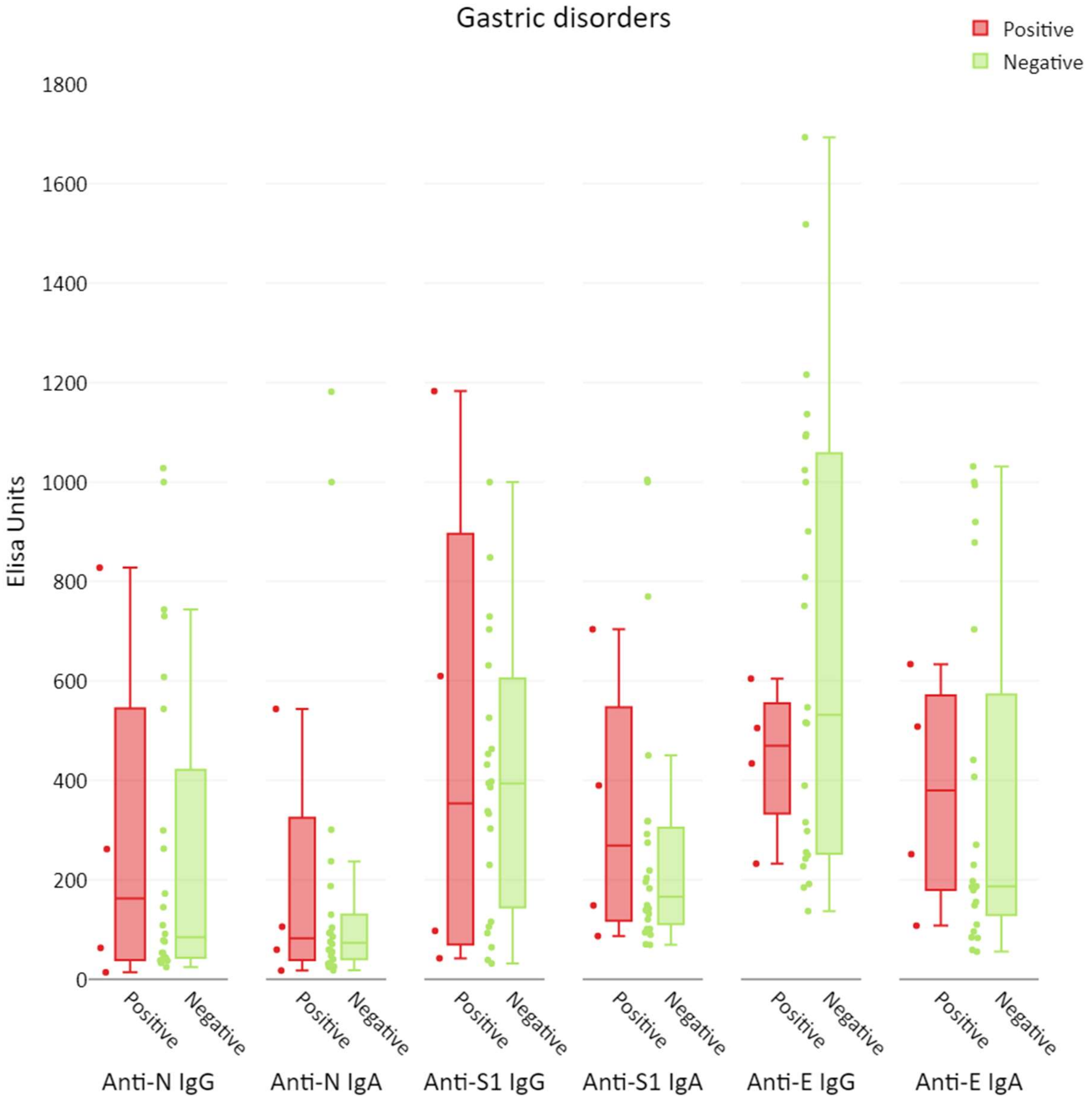

Figure S3. Gastric disorders during infection as a poor predictor of SARS-CoV-2 structural protein-specific antibodies induction. Samples from patients after infection were grouped according to observed (positive) or not observed (negative) breathing difficulties. All samples were tested by ELISA on SARS-CoV-2 protein-covered plates, and normalized using reference serum (as described by Miura et al. [1,2]); normalized ELISA units are presented, vertical lines in the boxes – median, boxes – values within 2nd and 3rd quartile, whiskers – SD, dots at the side of each box – real distribution of individual reads.

Supplementary Tables

Table S1.

Healthy

Sample	Sample Date	Anty-N IgG	Anty-N IgA	Anty-S1 IgG	Anty-S1 IgA	Anty-E IgG	Anty-E IgA	Age	Gender	Workplace	Temperature Over 38	Temperature over 37	Cough	Breath difficulties	Runny nose	Smell and taste disorders	Status	Date Status	Number of Symptoms	Sample order	Course of illness (COVID only)
179	24.04.2020	23,36	23,81	165,34	332,51	380,93	139,43	76	Male	Health Service	Negative	Positive	Positive	Negative	Positive	Negative	Healthy	Epidemic	5	A	
180	24.04.2020	22,20	11,16	180,24	85,46	159,73	32,98	No_Data	No_Data	No_Data	No_Data	No_Data	No_Data	No_Data	No_Data	No_Data	Healthy	Epidemic	0	A	
181	24.04.2020	21,03	16,87	265,64	113,95	388,67	89,46	35	Female	Health Service	Positive	Positive	Positive	Negative	Negative	Positive	Healthy	Epidemic	6	A	
182	24.04.2020	29,21	201,88	117,18	162,08	136,50	202,40	29	Male	Health Service	Negative	Negative	Negative	Negative	Negative	Negative	Healthy	Epidemic	0	A	
183	24.04.2020	19,86	26,79	349,80	117,88	152,23	112,44	47	Female	Health Service	Negative	Negative	Negative	Negative	Negative	Negative	Healthy	Epidemic	0	A	
184	24.04.2020	32,71	23,07	704,82	98,72	410,45	153,92	24	Male	Health Service	Negative	Positive	Positive	Positive	Positive	Negative	Healthy	Epidemic	5	A	
185	24.04.2020	78,27	15,63	66,04	94,30		66,97	41	Female	Health Service	Negative	Negative	Positive	Negative	Positive	Negative	Healthy	Epidemic	2	A	
186	24.04.2020	25,70	21,33	338,63	115,42	689,25	155,92	31	Female	Health Service	Negative	Negative	Negative	Negative	Negative	Negative	Healthy	Epidemic	0	A	
187	24.04.2020	25,70	18,35	506,21	201,87	157,79	164,42	33	Female	Health Service	Positive	Positive	Negative	Negative	Positive	Positive	Healthy	Epidemic	5	A	
188	24.04.2020	17,52	365,33	226,42	273,58	158,76	250,37	53	No_Data	Health Service	Negative	Positive	Negative	Negative	Negative	Negative	Healthy	Epidemic	1	A	
189	24.04.2020	24,53	59,28	163,60	235,27	334,95	272,86	36	Male	Health Service	Negative	Negative	Negative	Negative	Negative	Negative	Healthy	Epidemic	0	A	
190	24.04.2020	17,52	21,58	223,19	103,14	77,44	340,33	37	Male	Army	Negative	Negative	Negative	Negative	Negative	Negative	Healthy	Epidemic	0	A	
191	24.04.2020	36,21	29,02	65,04	132,61	193,37	253,37	No_Data	No_Data	No_Data	No_Data	No_Data	No_Data	No_Data	No_Data	No_Data	Healthy	Epidemic	0	A	
192	24.04.2020	23,36	17,36	302,38	87,92	464,42	58,97	52	Female	Health Service	Negative	Positive	Positive	Negative	Positive	Negative	Healthy	Epidemic	4	A	
193	24.04.2020	24,53	16,87	110,97	84,48	308,57	87,46	41	No_Data	Negative	Negative	Negative	Negative	Negative	Negative	Negative	Healthy	Epidemic	0	A	
194	24.04.2020	30,37	17,61	578,95	120,33	804,45	88,46	49	Female	Health Service	Negative	Negative	Negative	Negative	Negative	Negative	Healthy	Epidemic	0	A	
195	24.04.2020	22,20	25,79	368,17	169,94	721,44	178,41	34	Male	Health Service	Positive	Positive	Positive	Negative	Positive	Negative	Healthy	Epidemic	5	A	
196	24.04.2020	16,36	667,41	190,67	96,27	69,70	296,85	29	Female	Health Service	Negative	Negative	Negative	Negative	Negative	Negative	Healthy	Epidemic	0	A	
197	24.04.2020	19,86	22,57	358,74	117,88	182,72	86,46	23	Female	Office	Negative	Positive	Negative	Negative	Positive	Negative	Healthy	Epidemic	4	A	
198	24.04.2020	25,70	20,34	639,28	138,51	416,02	175,91	47	Female	Office	Negative	Positive	Positive	Negative	Positive	Negative	Healthy	Epidemic	4	A	
199	24.04.2020	25,70	57,79	276,07	130,65	180,78	103,45	40	Female	Researcher	Negative	Negative	Negative	Negative	Positive	Negative	Healthy	Epidemic	2	A	
199B	09.10.2020	28,04	48,61	404,17	71,71	224,59	79,96	40	Female	Researcher	Negative	Negative	Negative	Negative	Positive	Negative	Healthy	Epidemic	2	B	
200	24.04.2020	19,86	24,80	113,70	204,32	85,19	387,31	No_Data	No_Data	No_Data	No_Data	No_Data	No_Data	No_Data	No_Data	No_Data	Healthy	Epidemic	0	A	
201	24.04.2020	19,86	10,66	228,15	71,71	121,97	78,96	42	Female	Health Service	Negative	Negative	Negative	Negative	Negative	Negative	Healthy	Epidemic	0	A	
202	24.04.2020	26,87	22,32	530,29	115,42	131,41	125,44	37	No_Data	No_Data	Negative	Negative	Negative	Negative	Negative	Negative	Healthy	Epidemic	0	A	
203	28.04.2020	18,69	14,14	319,51	66,80	128,03	61,97	No_Data	Female	Health Service	Negative	Negative	Negative	Negative	Negative	Negative	Healthy	Epidemic	0	A	
204	28.04.2020	22,20	33,48	302,63	144,40	321,88	216,89	50	Female	Health Service	Negative	Positive	Negative	Negative	Negative	Negative	Healthy	Epidemic	3	A	
205	28.04.2020	15,19	33,98	134,06	125,25	74,54	261,87	45	Female	Health Service	Negative	Negative	Negative	Negative	Negative	Negative	Healthy	Epidemic	2	A	
206	28.04.2020	30,37	74,40	323,73	427,80		423,79	67	Female	No_Data	Negative	Negative	Negative	Negative	Negative	Negative	Healthy	Epidemic	0	A	
207	28.04.2020	23,36	37,95	43,20	102,65	321,88	152,42	56	Female	Health Service	Negative	Negative	Negative	Negative	Negative	Negative	Healthy	Epidemic	0	A	
208	28.04.2020	42,06	49,85	43,20	565,32	366,41	77,96	35	Female	Health Service	Negative	Negative	Negative	Negative	Negative	Negative	Healthy	Epidemic	0	A	
209	28.04.2020	21,03	82,34	61,57	210,22	1275,90	219,39	57	No_Data	Health Service	Negative	Negative	Negative	Negative	Negative	Negative	Healthy	Epidemic	0	A	
210	28.04.2020	16,36	53,32	50,15	176,33	223,14	66,47	46	Female	Health Service	Negative	Positive	Positive	Negative	Negative	Negative	Healthy	Epidemic	4	A	
211	28.04.2020	31,54	106,65	51,14	276,77	294,77	472,76	48	Female	Health Service	Negative	Negative	Negative	Negative	Negative	Negative	Healthy	Epidemic	0	A	
212	28.04.2020	29,21	47,37	69,02	468,57	843,66	109,95	72	Female	Health Service	Negative	Negative	Negative	Negative	Negative	Negative	Healthy	Epidemic	0	A	
213	28.04.2020	21,03	24,80	47,67	43,47	317,04	26,99	No_Data	No_Data	No_Data	No_Data	No_Data	No_Data	No_Data	No_Data	No_Data	Healthy	Epidemic	0	A	
214	28.04.2020	28,04	122,27	51,14	284,63	578,41	514,24	51	Female	Airport	Negative	Negative	Positive	Negative	Negative	Negative	Healthy	Epidemic	1	A	
215	28.04.2020	16,36	49,60	52,63	105,35	444,82	85,96	34	Male	Health Service	Negative	Negative	Negative	Negative	Negative	Negative	Healthy	Epidemic	0	A	
216	29.04.2020	21,03	49,60	45,18	115,91	333,01	147,43	No_Data	Female	Health Service	Negative	Negative	Positive	Negative	Positive	Negative	Healthy	Epidemic	2	A	
217	29.04.2020	11,68	15,38	48,66	70,48	244,92	38,98	No_Data	No_Data	No_Data	No_Data	No_Data	No_Data	No_Data	No_Data	No_Data	Healthy	Epidemic	0	A	
218	29.04.2020	18,69	33,48	62,56	119,84	436,59	90,95	38	Male	Health Service	Positive	Positive	Negative	Positive	Positive	Negative	Healthy	Epidemic	3	A	
219	29.04.2020	15,19	49,36	51,64	100,20	260,89	123,94	38	Female	Health Service	Negative	Negative	Negative	Negative	Negative	Negative	Healthy	Epidemic	0	A	
220	29.04.2020	36,21	41,91	62,56	46,91	395,45	21,99	No_Data	Female	No_Data	Negative	Negative	Negative	Negative	Negative	Negative	Healthy	Epidemic	0	A	
221	29.04.2020	21,03	85,57	43,69	137,03	545,98	313,84	60	Male	Health Service	Negative	Negative	Negative	Negative	Positive	Negative	Healthy	Epidemic	1	A	
222	29.04.2020	24,53	81,35	71,50	131,63		124,44	46	Female	Health Service	Negative	Negative	Negative	Negative	Negative	Negative	Healthy	Epidemic	0	A	
223	29.04.2020	29,21	51,34	61,07	142,19	905,13	229,39	No_Data	Female	Health Service	Negative	Negative	Negative	Negative	Negative	Negative	Healthy	Epidemic	0	A	
224	29.04.2020	30,37	35,96	64,55	89,64	562,92	76,96	No_Data	No_Data	No_Data	No_Data	No_Data	No_Data	No_Data	No_Data	No_Data	Healthy	Epidemic	0	A	
225	29.04.2020	17,52	38,94	52,14	204,81	620,52	155,42	No_Data	No_Data	No_Data	No_Data	No_Data	No_Data	No_Data	No_Data	No_Data	Healthy	Epidemic	0	A	
226	29.04.2020	30,37	41,17	67,53	102,65	1241,05	72,96	64	Female	Health Service	Negative	Negative	Negative	Negative	Negative	Negative	Healthy	Epidemic	0	A	
227	29.04.2020	29,21	40,67	55,11	113,21	331,56	206,90	55	Male	Health Service	Negative	Negative	Negative	Negative	Negative	Negative	Healthy	Epidemic	0	A	
228	30.04.2020	51,40	43,15	53,62	151,28	590,51	260,87	24	Female	Health Service	Negative	Negative	Negative	Negative	Negative	Negative	Healthy	Epidemic	1	A	
229	30.04.2020	22,20	67,96	60,58	165,77	542,59	232,88	60	Female	Health Service	Negative	Negative	Negative	Negative	Negative	Negative	Healthy	Epidemic	0	A	
230	30.04.2020	15,19	23,31	33,27	47,15	143,27	44,98	23	Female	Health Service	Negative	Negative	Negative	Negative	Negative	Negative	Healthy	Epidemic	0	A	
231	30.04.2020	12,85	42,16	42,20	124,02	621,97	125,94	44	Female	No_Data	Positive	Positive	Positive	Negative	Positive	Negative	Healthy	Epidemic	5	A	
232	05.05.2020	21,03	42,41	51,64	71,71	388,67	78,96	52	Male	Health Service	Negative	Negative	Negative	Negative	Negative	Negative	Healthy	Epidemic	0	A	
233	05.05.2020	22,20	58,53	53,13	127,95	407,55	149,93	58	Female	Health Service	Negative	Negative	Negative	Negative	Negative	Negative	Healthy	Epidemic	0	A	
234	08.05.2020	25,70	89,29	171,30	117,06	351,65	140,93	34	Male	Health Service	Negative	Negative	Negative	Negative	Negative	Negative	Healthy	Epidemic	0	A	
235	07.05.2020	14,02	13,64	118,67	110,02	237,66	110,44	32	Male	Office	Negative	Negative	Negative	Negative	Negative	Negative	Healthy	Epidemic	0	A	
236	07.05.2020	23,36	14,38	168,82	114,93	553,73	102,45	31	Female	Office	Negative	Negative	Negative	Negative	Negative	Negative	Healthy	Epidemic	1	A	
237	07.05.2020	22,20	23,81	246,28	127,70	339,30	117,44	30	Male	Office	Negative	Negative	Negative	Negative	Negative	Negative	Healthy	Epidemic	0	A	
238	07.05.2020	18,69	8,68	539,72	93,32	410,94	78,96	67	Female	Pensioner	Negative	Positive	Positive	Positive	Negative	Negative	Healthy	Epidemic	4	A	
239	07.05.2020	46,73	210,32	701,09	133,10	785,58	111,44	26	Female	Researcher	Positive	Positive	Positive	Positive	Positive	Negative	Healthy	Epidemic	6	A	
240	07.05.2020	22,20	42,66	3,97	407,17	817,52	445,78	37	Female	Researcher	Positive	Positive	Negative	Negative	Negative	Negative	Healthy	Epidemic	3	A	
241	07.05.2020	14,02	14,88	98,31	71,71	532,43	66,47	29	Female	Researcher	Negative	Negative	Negative	Negative	Positive	Negative	Healthy	Epidemic	2	A	
242	07.05.2020	28,04	25,30	314,80	185,17	611,33	235,38	30	Female	Researcher	Negative	Negative	Positive	Negative	Positive	Negative	Healthy	Epidemic	2	A	
243	07.05.2020	18,69	9,92	157,40	72,69	515,97	74,96	26	Male	Researcher	Positive	Positive	Negative	Positive	Positive	Negative	Healthy	Epidemic	5	A	
243B	13.10.2020	28,04	13,89	313,80	40,28	162,63	36,98	26	Male	Researcher	Positive	Positive	Negative	Positive	Positive	Negative	Healthy	Epidemic	5	B	
244	07.05.2020	19,86	53,32	498,76	108,06	589,55	84,96	29	Male	Office	Negative	Negative	Negative	Negative	Negative	Negative	Healthy	Epidemic	0	A	
245	07.05.2020	14,02	48,12	58,59	244,11	395,45	309,35	31	Female	Researcher	Negative	Negative	Negative	Negative	Negative	Negative	Healthy	Epidemic	0	A	
246	07.05.2020	9,33	23,56	236,35	189,59	376,57	500,75	29	Male	Researcher	Positive	Positive	Negative	Negative	Negative	Negative	Healthy	Epidemic	2	A	
247	08.05.2020	7,01	6,20	99,80	56,97	188,29	56,47	No_Data	No_Data	No_Data	No_Data	No_Data	No_Data	No_Data	No_Data	No_Data	Healthy	Epidemic	0	A	
248	08.05.2020	14,02	25,05	141,01	141,94	318,01	293,85	49	Male	Health Service	Negative	Negative	Positive	Negative	Positive	Negative	Healthy	Epidemic	2	A	
248B	10.06.2020	25,70	53,32	972,69	160,85	299,94	203,40	49	Male	Health Service	Negative	Negative	Positive	Negative	Positive	Negative	Healthy	Epidemic	2	B	
248C	14.10.2020	28,04	27,78	431,98	164,05	380,45	117,94	49	Male	Health Service	Negative	Negative	Positive	Negative	Positive	Negative	Healthy	Epidemic	2	C	
249	11.05.2020	11,68	5,70	95,83	63,36	235,72	46,98	No_Data	No_Data	No_Data	No_Data	No_Data	No_Data	No_Data	No_Data	No_Data	Healthy	Epidemic	0	A	
250	13.05.2020	18,69	26,54	170,80	176,33	687,80	176,41	59	Female	Education	Negative	Negative	Positive	Positive	Negative	Negative	Healthy	Epidemic	3	A	
251	11.05.2020	10,51	22,07	88,88	217,58	315,59	323,84	50	Female	Health Service	Negative	Negative	Negative	Negative	Negative	Negative	Healthy	Epidemic	0	A	
252	19.05.2020	14,02	13,39	55,11	77,11	236,21	96,95	38	Male	Health Service	Negative	Negative	Negative	Negative	Negative	Negative	Healthy	Epidemic	0	A	
253	13.05.2020	10,51	32,49	102,78	422,89	494,19	463,27	33	Male	Office	Negative	Positive	Positive	Negative	Positive	Negative	Healthy	Epidemic	5	A	
254	13.05.2020	30,37	13,64	144,49	82,51	424,49	125,94	21	Male	Student	Negative	Negative	Positive	Negative	Positive	Negative	Healthy	Epidemic	4	A	
255	11.05.2020	17,52	14,14	171,30	103,63	781,22	114,94	33	Female	Office	Negative	Negative	Negative	Negative	Positive	Negative	Healthy	Epidemic	3	A	
256	11.05.2020	29,21	13,89	205,06	105,11	370,28	170,41	25	Male	Public Transport Driver	Negative	Negative	Positive	Negative	Positive	Negative	Healthy	Epidemic	3	A	
257	11.05.2020	16,36	15,63	940,91	154,22	634,08	204,40	65	Female	Researcher	Positive	Positive	Positive	Negative	Negative	Negative	Healthy	Epidemic	3	A	
258	11.05.2020	23,36	18,35	264,65	134,09	344,63	123,94	45	Female	Office	Negative	Negative	Negative	Negative	Negative	Negative	Healthy	Epidemic	1	A	

259	11.05.2020	32,71	42,16	560,08	186,15	768,64	418,29	50	Male	Builder	Positive	Positive	Positive	Positive	Negative	Negative	Healthy	Epidemic	5	A	
260	11.05.2020	17,52	22,57	240,32	93,81	334,95	103,95	43	Female	Sales Manager	Positive	Positive	Negative	Negative	Positive	Positive	Healthy	Epidemic	5	A	
261	11.05.2020	47,90	24,55	857,00	269,65	308,33	354,82	58	Female	Health Service	Negative	Positive	Positive	Negative	Positive	Positive	Healthy	Epidemic	6	A	
262	11.05.2020	15,19	20,34	125,12	177,31	316,07	707,15	26	Female	Office	Negative	Positive	Positive	Negative	Positive	Negative	Healthy	Epidemic	4	A	
263	11.05.2020	29,21	48,86	637,04	278,24	573,57	735,13	32	Male	Office	Positive	Positive	Positive	Negative	Positive	Negative	Healthy	Epidemic	4	A	
264	11.05.2020	85,28	40,18	143,00	199,90	949,66	376,81	36	Male	No_Data	Negative	Negative	Negative	Negative	Negative	Negative	Healthy	Epidemic	0	A	
265	11.05.2020	26,87	20,09	207,05	67,78	818,73	93,95	44	Female	Researcher	Positive	Positive	Positive	Negative	Positive	Negative	Healthy	Epidemic	4	A	
265B	08.10.2020	28,04	34,72	519,36	77,60	538,24	97,95	44	Female	Researcher	Positive	Positive	Positive	Negative	Positive	Negative	Healthy	Epidemic	4	B	
266	11.05.2020	26,87	49,60	391,26	385,07	604,55	763,62	64	Female	Pensioner	Negative	Negative	Negative	Negative	Negative	Negative	Healthy	Epidemic	0	A	
267	11.05.2020	79,44	71,68	123,14	206,78	887,22	355,32	27	Male	Unemployed	Negative	Negative	Negative	Negative	Negative	Negative	Healthy	Epidemic	0	A	
268	11.05.2020	26,87	29,27	99,30	119,11	529,04	280,36	60	Female	Office	Negative	Negative	Positive	Negative	Negative	Negative	Healthy	Epidemic	2	A	
269	13.05.2020	75,93	186,26	153,92	164,29	463,21	399,80	50	Male	Education	Negative	Negative	Negative	Negative	Negative	Negative	Healthy	Epidemic	0	A	
270	13.05.2020	12,85	16,87	73,98	80,30	485,16	131,43	59	Male	Pensioner	Negative	Negative	Positive	Positive	Positive	Negative	Healthy	Epidemic	3	A	
271	13.05.2020	22,20	14,38	182,22	36,35	440,95	75,46	19	Male	Student	Negative	Positive	Positive	Negative	Positive	Negative	Healthy	Epidemic	4	A	
272	08.05.2020	21,03	65,23	201,09	157,42	587,37	287,86	29	Female	Police	Negative	Negative	Negative	Negative	Negative	Negative	Healthy	Epidemic	0	A	
273	13.05.2020	15,19	26,29	95,83	113,21	232,58	133,93	32	Male	Police	Negative	Negative	Negative	Positive	Negative	Negative	Healthy	Epidemic	1	A	
274	13.05.2020	15,19	19,59	84,91	41,99	282,43	94,45	51	Male	Office	Positive	Positive	Positive	Positive	Negative	Negative	Healthy	Epidemic	3	A	
275	13.05.2020	25,70	24,80	300,89	104,13	623,91	207,40	28	Female	Education	Negative	Negative	Positive	Negative	Positive	Negative	Healthy	Epidemic	4	A	
276	13.05.2020	21,03	40,43	129,59	94,06	350,92	313,84	51	Male	Medical Sales representative	Positive	Positive	Negative	Positive	Positive	Negative	Healthy	Epidemic	6	A	
277	19.05.2020	25,70	30,01	107,25	78,34	199,42	128,94	31	Male	Health Service	Negative	Positive	Positive	Positive	Positive	Negative	Healthy	Epidemic	6	A	
278	09.05.2020	19,86	34,72	145,98	195,48	583,25	234,88	54	Male	Office	Positive	Positive	Positive	Positive	Positive	Negative	Healthy	Epidemic	6	A	
279	09.05.2020	14,02	25,05	115,19	74,90	613,59	137,43	49	Female	Health Service	Negative	Negative	Negative	Negative	Negative	Negative	Healthy	Epidemic	0	A	
280	19.05.2020	38,55	43,40	387,79	757,37	1417,23	1402,80	No_Data	Male	Health Service	Negative	Negative	Negative	Negative	Negative	Negative	Healthy	Epidemic	0	A	
281	19.05.2020	23,36	33,73	214,50	97,25	1095,84	304,35	32	Male	Health Service	Negative	Negative	Negative	Negative	Negative	Negative	Healthy	Epidemic	0	A	
282	11.05.2020	38,55	60,76	152,93	179,52	569,70	464,27	No_Data	No_Data	No_Data	No_Data	No_Data	No_Data	No_Data	No_Data	No_Data	Healthy	Epidemic	0	A	
283	11.05.2020	23,36	106,15	177,76	120,09	347,21	780,61	No_Data	No_Data	No_Data	No_Data	No_Data	No_Data	No_Data	No_Data	No_Data	Healthy	Epidemic	0	A	
284	12.05.2020	16,36	36,71	124,13	106,34	381,41	310,84	33	Male	Office	Negative	Negative	Negative	Negative	Negative	Negative	Healthy	Epidemic	0	A	
285	12.05.2020	38,55	26,54	407,65	115,18	731,36	348,33	46	Female	Lecturer	Positive	Positive	Positive	Negative	Negative	Negative	Healthy	Epidemic	4	A	
286	13.05.2020	25,70	29,76	443,40	91,36	362,86	251,37	35	Female	Office	Negative	Negative	Negative	Negative	Negative	Negative	Healthy	Epidemic	0	A	
287	14.05.2020	30,37	31,50	186,69	117,39	879,48	312,34	45	Female	Office	Negative	Positive	Negative	Negative	Positive	Negative	Healthy	Epidemic	2	A	
288	14.05.2020	35,05	60,76	289,97	228,39	747,82	1079,46	45	Male	Production hall	Negative	Negative	Negative	Negative	Negative	Negative	Healthy	Epidemic	0	A	
289	14.05.2020	11,68	44,64	52,63	62,62	278,48	197,90	45	Female	Health Service	Negative	Positive	Positive	Negative	Positive	Negative	Healthy	Epidemic	5	A	
290	14.05.2020	51,40	29,27	347,07	101,42	816,88	267,37	44	Male	Office	Negative	Negative	Negative	Negative	Negative	Negative	Healthy	Epidemic	0	A	
291	14.05.2020	54,91	108,13	290,47	292,73	580,11	489,26	44	Female	Office	Negative	Positive	Negative	Negative	Positive	Negative	Healthy	Epidemic	3	A	
292	14.05.2020	26,87	24,80	414,60	136,54	984,03	98,95	22	Female	Student	Negative	Negative	Negative	Negative	Negative	Negative	Healthy	Epidemic	0	A	
293	14.05.2020	32,71	32,74	492,80	140,96	169,41	121,94	61	Female	Pensioner	Negative	Negative	Positive	Negative	Negative	Negative	Healthy	Epidemic	2	A	
294	14.05.2020	31,54	65,97	487,09	253,44		276,36	32	Female	Office	Negative	Negative	Negative	Negative	Negative	Negative	Healthy	Epidemic	0	A	
295	30.04.2020	23,36	38,19	444,39	122,79	832,77	131,43	65	Male	Health Service	Negative	Negative	Negative	Negative	Negative	Negative	Healthy	Epidemic	0	A	
296	14.05.2020	25,70	61,01	283,27	214,15	928,85	440,28	No_Data	No_Data	No_Data	No_Data	No_Data	No_Data	No_Data	No_Data	No_Data	Healthy	Epidemic	0	A	
297	14.05.2020	39,72	81,85	427,01	329,57		453,27	53	Female	Office	Negative	Negative	Negative	Negative	Negative	Negative	Healthy	Epidemic	0	A	
298	14.05.2020	59,58	69,44	227,90	109,53	296,47	117,94	29	Female	Office	Negative	Positive	Positive	Negative	Positive	Negative	Healthy	Epidemic	5	A	
299	15.05.2020	21,03	63,00	197,86	248,04	383,11	293,35	49	Female	Health Service	Negative	Negative	Negative	Negative	Positive	Negative	Healthy	Epidemic	1	A	
300	15.05.2020	40,89	44,15	364,45	230,84	437,56	329,84	42	Male	Office	Negative	Negative	Negative	Negative	Negative	Negative	Healthy	Epidemic	1	A	
301	15.05.2020	49,07	115,58	759,43	648,82	156,82	809,10	48	Male	Office	Negative	Negative	Negative	Negative	Negative	Negative	Healthy	Epidemic	1	A	
302	15.05.2020	30,37	55,56	454,07	218,07	655,13	379,31	40	Female	Artitst	Negative	Positive	Negative	Negative	Negative	Negative	Healthy	Epidemic	3	A	
303	15.05.2020	33,88	32,24	334,66	101,18		189,91	36	Female	Office	Negative	Positive	Positive	Positive	Negative	Negative	Healthy	Epidemic	5	A	
304	15.05.2020	24,53	44,15	337,39	195,48	433,20	257,37	38	Male	Office	Negative	Negative	Positive	Negative	Positive	Negative	Healthy	Epidemic	4	A	
305	15.05.2020	23,36	39,19	298,91	140,47	353,58	221,89	46	Male	Public Transport Driver	Negative	Negative	Negative	Negative	Negative	Negative	Healthy	Epidemic	0	A	
306	15.05.2020	18,69	35,22	238,08	174,36	228,70	244,38	39	Female	Office	Negative	Negative	Positive	Negative	Negative	Negative	Healthy	Epidemic	2	A	
307	15.05.2020	16,36	61,01	289,47	250,00	179,82	499,25	26	Female	Shop	Negative	Positive	Negative	Negative	Negative	Negative	Healthy	Epidemic	3	A	
308	15.05.2020	30,37	148,31	222,94	435,66		906,55	43	Male	Shop	Negative	Negative	Negative	Negative	Negative	Negative	Healthy	Epidemic	0	A	
309	15.05.2020	63,08	107,64		299,61		1240,88	46	Male	Technical employee	Negative	Positive	Positive	Negative	Positive	Negative	Healthy	Epidemic	3	A	
310	15.05.2020	33,88	41,17	249,01	157,17	1408,52	212,89	37	Female	Office	Negative	Negative	Negative	Negative	Negative	Negative	Healthy	Epidemic	0	A	
311	15.05.2020	9,35	58,53	206,06	204,32	146,18	200,40	41	Female	Shop	Negative	Negative	Negative	Negative	Negative	Negative	Healthy	Epidemic	0	A	
312	15.05.2020	39,72	112,10	481,88	261,79	109,39	473,26	36	Female	Shop	Negative	Negative	Positive	Negative	Positive	Negative	Healthy	Epidemic	5	A	
313	15.05.2020	39,72	47,62	570,26	214,64	123,91	224,39	42	Female	Office	Negative	Negative	Positive	Negative	Negative	Negative	Healthy	Epidemic	3	A	
314	15.05.2020	16,36	78,37	349,30	352,65	126,33	353,32	45	Female	Office	Negative	Positive	Positive	Negative	Positive	Negative	Healthy	Epidemic	4	A	
315	15.05.2020	18,69	61,01	284,01	129,67	422,31	271,86	28	Male	Production hall	Negative	Positive	Negative	Negative	Positive	Negative	Healthy	Epidemic	3	A	
316	15.05.2020	31,54	43,15	176,02	331,53	350,44	334,33	28	Male	Production hall	Negative	Positive	Negative	Negative	Positive	Negative	Healthy	Epidemic	3	A	
317	14.05.2020	36,21	169,15	472,69	530,94	604,55	698,65	41	Male	Production hall	Negative	Negative	Negative	Negative	Negative	Negative	Healthy	Epidemic	0	A	
318	14.05.2020	12,85	48,61	149,45	209,72	308,81	281,86	43	Female	Health Service	Negative	Negative	Negative	Negative	Positive	Negative	Healthy	Epidemic	3	A	
319	14.05.2020	66,59	67,96	283,52	191,55	521,30	184,91	28	Male	Office	Positive	Positive	Positive	Positive	Negative	Positive	Healthy	Epidemic	5	A	
320	14.05.2020	30,37	45,63	642,01	195,48	840,76	112,94	38	Male	Office	Negative	Negative	Negative	Negative	Negative	Negative	Healthy	Epidemic	0	A	
321	14.05.2020	50,62	116,82	1016,63	443,52		658,17	69	Female	No_Data	Positive	Positive	Positive	Positive	Positive	Negative	Healthy	Epidemic	5	A	
322	14.05.2020	50,23	76,88	148,46	308,94	340,76	372,81	39	Female	Office	Negative	Positive	Positive	Negative	Negative	Negative	Healthy	Epidemic	2	A	
323	18.05.2020	23,36	67,96	186,69	324,17	277,83	373,31	27	Male	Health Service	Negative	Negative	Negative	Negative	Negative	Negative	Healthy	Epidemic	1	A	
324	13.05.2020	22,20	106,65	331,68	802,06	279,77	838,58	69	Male	Health Service	Negative	Negative	Positive	Negative	Negative	Negative	Healthy	Epidemic	1	A	
325	19.05.2020	39,72	64,98	422,54	137,52	472,41	143,93	48	Female	Laboratory	Negative	Negative	Positive	Positive	Negative	Negative	Healthy	Epidemic	4	A	
326	16.05.2020	57,24	43,65	429,99	129,17	644,72	127,94	34	Female	Office	Negative	Negative	Negative	Negative	Negative	Negative	Healthy	Epidemic	0	A	
327	16.05.2020	39,72	60,52	990,07	394,40	680,54	147,93	30	Female	Office	Negative	Negative	Negative	Negative	Negative	Negative	Healthy	Epidemic	0	A	
328	16.05.2020	29,21	71,92	122,14	328,09	233,30	752,62	31	Male	Office	Negative	Negative	Positive	Negative	Negative	Negative	Healthy	Epidemic	3	A	
329	19.05.2020	29,21	75,40	665,84	1218,07		484,26	50	Male	Health Service	Negative	Negative	Negative	Negative	Negative	Negative	Healthy	Epidemic	0	A	
330	19.05.2020	39,72	68,45	282,27	239,19	868,83	394,80	53	Female	Health Service	Negative	Negative	Negative	Negative	Negative	Negative	Healthy	Epidemic	1	A	
331	19.05.2020	30,37	78,37	612,71	299,61	942,88	294,35	49	Female	Health Service	Negative	Negative	Negative	Negative	Negative	Negative	Healthy	Epidemic	0	A	
332	19.05.2020	40,89	54,07	685,20	212,67	900,29	175,41	47	Female	Health Service	Negative	Negative	Negative	Negative	Negative	Negative	Healthy	Epidemic	0	A	
333	19.05.2020	32,71	128,47	216,98	307,96	250,24	813,59	30	Male	Health Service	Negative	Negative	Positive	Negative	Positive	Negative	Healthy	Epidemic	2	A	
334	19.05.2020	29,21	23,81	191,16	61,89	319,46	52,47	37	Female	Health Service	Negative	Negative	Negative	Negative	Negative	Negative	Healthy	Epidemic	0	A	
335	19.05.2020	26,87	65,97	275,07	242,63	401,74	175,41	53	Female	Health Service	Negative	Positive	Negative	Negative	Negative	Negative	Healthy	Epidemic	3	A	
336	19.05.2020	74,77	38,19	146,97	88,90	7,74	76,96	50	Female	Health Service	Negative	Negative	Positive	Negative	Positive	Negative	Healthy	Epidemic	2	A	
337	22.05.2020	31,54	78,37	281,03	201,38	238,63	308,35	35	Female	Office	Positive	Positive	Positive	Positive	Negative	Negative	Healthy	Epidemic	5	A	
338	22.05.2020	67,76	49,11	439,42	206,29	175,22	119,44	27	Female	Health Service	Negative	Negative	Positive	Negative	Negative	Negative	Healthy	Epidemic	2	A	
339	22.05.2020	26,87	43,65	672,79	123,28	203,78	62,97	40	Male	Health Service	Negative	Negative	Negative	Negative	Negative	Negative	Healthy	Epidemic	0	A	
340	22.05.2020	49,07	325,89	365,94	434,18	651,02	568,22	52	Male	Health Service	Negative	Negative	Negative	Negative	Negative	Negative	Healthy	Epidemic	0	A	
341	22.05.2020	16,36	30,75	145,48	141,45	136,98	57,97	53	Female	No_Data	Negative	Negative	Negative	Negative	Negative	Negative	Healthy	Epidemic	0	A	
342	22.05.2020	30,37	81,85	312,31	410,12	758,47	753,62	56	Female	Health Service	Negative	Negative	Negative	Negative	Negative	Negative	Healthy	Epidemic	0	A	
343	22.05.2020	22,20	94,25	113,21	364,93	146,66	296,35	47	Female	Health Service	Negative	Negative	Negative	Negative	Negative	Negative	Healthy	Epidemic	0	A	
344	22.05.2020	22,20	54,07	167,83	135,56	290,42	111,44	25	Female	Health Service	Negative	Negative	Negative	Negative	Negative	Negative	Healthy	Epidemic	0	A	
345	22.05.2020	32,71	169,15	430,49	578,59	326,23	431,28	55	Male	Health Service	Negative	Negative	Negative	Negative	Negative	Negative	Healthy	Epidemic	0	A	

Sample	Sample Date	Anty-N IgG	Anty-N IgA	Anty-S1 IgG	Anty-S1 IgA	Anty-E IgG	Anty-E IgA	Age	Gender	Workplace	Temperature Over 38	Temperature over 37	Cough	Breath difficulties	Runny nose	Smell and taste disorders	Status	Date Status	Number of Symptoms	Sample order	Course of illness (COVID only)
346	22.05.2020	22,20	67,46	261,67	276,52	232,82	122,44	53	Female	Health Service	Negative	Negative	Negative	Negative	Negative	Negative	Healthy	Epidemic	0	A	
347	22.05.2020	32,71	83,33	1054,62	487,23		386,31	42	Female	Health Service	Negative	Negative	Positive	Negative	Positive	Negative	Healthy	Epidemic	4	A	
348	22.05.2020	29,21	36,71	576,46	160,61	169,89	92,95	51	Female	Health Service	Negative	Negative	Negative	Negative	Negative	Negative	Healthy	Epidemic	0	A	
349	22.05.2020	32,71	38,19	496,03	110,02	853,82	114,44	53	Male	Health Service	Negative	Positive	Positive	Positive	Positive	Negative	Healthy	Epidemic	6	A	
350	22.05.2020	16,36	83,33	337,14	183,69	82,28	204,40	27	Female	Health Service	Negative	Negative	Negative	Negative	Positive	Negative	Healthy	Epidemic	1	A	
351	22.05.2020	25,70	54,56	492,55	161,10	150,53	250,87	34	Male	Health Service	Negative	Negative	Negative	Negative	Negative	Negative	Healthy	Epidemic	0	A	
352	22.05.2020	24,53	53,57	141,51	204,32	49,85	220,39	34	Male	Health Service	Negative	Negative	Positive	Negative	Positive	Negative	Healthy	Epidemic	4	A	
353	22.05.2020	23,36	100,69	178,25	291,26	68,73	110,94	45	Male	Health Service	Negative	Negative	Positive	Positive	Negative	Negative	Healthy	Epidemic	3	A	
354	22.05.2020	30,37	24,80	375,37	155,70	227,49	110,44	51	Female	Health Service	Negative	Negative	Negative	Negative	Negative	Negative	Healthy	Epidemic	0	A	
355	22.05.2020	29,21	93,25	724,43	234,28		265,87	43	Female	Health Service	Negative	Positive	Positive	Positive	Positive	Negative	Healthy	Epidemic	6	A	
356	22.05.2020	25,70	40,67	272,59	145,87	114,71	172,91	56	Male	Health Service	Negative	Negative	Negative	Negative	Negative	Negative	Healthy	Epidemic	0	A	
357	22.05.2020	23,36	49,60	270,11	196,46	43,56	214,39	42	Female	Health Service	Negative	Negative	Negative	Negative	Negative	Negative	Healthy	Epidemic	1	A	
358	22.05.2020	45,95	52,83	1098,31	234,28		533,48	45	Male	Health Service	Negative	Negative	Negative	Negative	Negative	Negative	Healthy	Epidemic	0	A	
359	22.05.2020	40,89	47,12	514,40	173,87	134,56	163,92	34	Male	Health Service	Negative	Negative	Negative	Negative	Negative	Negative	Healthy	Epidemic	0	A	
360	22.05.2020	37,38	45,14	370,90	114,93	629,72	87,46	40	Female	Health Service	Negative	Negative	Negative	Negative	Negative	Negative	Healthy	Epidemic	0	A	
361	22.05.2020	29,21	49,60	456,80	131,63	64,86	199,40	26	Male	Health Service	Negative	Negative	Negative	Negative	Negative	Negative	Healthy	Epidemic	1	A	
362	22.05.2020	23,36	50,60	60,58	114,44	60,02	132,43	61	Male	Health Service	Negative	Negative	Negative	Negative	Negative	Negative	Healthy	Epidemic	0	A	
363	22.05.2020	26,87	58,04	285,50	110,02	152,47	616,19	58	Female	Health Service	Negative	Negative	Negative	Negative	Negative	Negative	Healthy	Epidemic	0	A	
364	22.05.2020	15,19	105,16	72,00	221,51	30,98	352,32	42	Male	Health Service	Positive	Positive	Positive	Negative	Negative	Negative	Healthy	Epidemic	4	A	
365	22.05.2020	18,69	63,00	183,71	195,48	66,31	248,38	No_Data	Male	Health Service	Positive	Positive	Positive	Negative	Negative	Negative	Healthy	Epidemic	3	A	
366	22.05.2020	17,52	17,36	186,69	42,24	49,37	49,48	59	Male	Health Service	Negative	Negative	Negative	Negative	Negative	Negative	Healthy	Epidemic	0	A	
367	22.05.2020	26,87	152,28	395,73	529,96	86,16		52	Male	Health Service	Negative	Negative	Negative	Negative	Negative	Negative	Healthy	Epidemic	0	A	
368	22.05.2020	33,88	137,40	541,21	584,48			49	Male	Health Service	Negative	Negative	Negative	Negative	Negative	Negative	Healthy	Epidemic	0	A	
369	22.05.2020	18,69	63,99	155,41	134,58	68,25	160,92	37	Female	Health Service	Negative	Negative	Negative	Negative	Negative	Negative	Healthy	Epidemic	0	A	
370	22.05.2020	24,53	49,11	189,67	195,97	82,28	130,43	43	Female	Health Service	Negative	Negative	Negative	Negative	Negative	Negative	Healthy	Epidemic	0	A	
371	22.05.2020	22,20	17,36	236,35	67,29	78,41	59,47	57	Female	Health Service	Negative	Negative	Negative	Negative	Negative	Negative	Healthy	Epidemic	0	A	
372	22.05.2020	25,70	47,62	202,58	166,50	45,50	246,38	56	Female	Health Service	Negative	Positive	Positive	Positive	Negative	Negative	Healthy	Epidemic	5	A	
373	22.05.2020	25,70	42,66	173,29	110,02	54,70	102,45	49	Female	Health Service	Negative	Negative	Negative	Negative	Negative	Negative	Healthy	Epidemic	0	A	
374	22.05.2020	17,52	27,28	96,82	89,88	47,92	287,86	35	Male	Health Service	Negative	Negative	Negative	Negative	Negative	Negative	Healthy	Epidemic	0	A	
375	22.05.2020	12,85	40,18	81,43	115,91	156,10	170,91	38	Male	Health Service	Negative	Negative	Negative	Negative	Negative	Negative	Healthy	Epidemic	0	A	
376	22.05.2020	17,52	46,13	106,26	161,59	210,79	761,62	32	Male	Health Service	Positive	Positive	Negative	Negative	Positive	Negative	Healthy	Epidemic	2	A	
377	22.05.2020	31,54	25,79	306,85	89,39	1166,51	112,94	51	Female	Health Service	Negative	Negative	Negative	Negative	Negative	Negative	Healthy	Epidemic	0	A	
378	22.05.2020	19,86	21,83	7,94	56,97	295,26	23,99	55	Female	Health Service	Positive	Positive	Positive	Negative	Positive	Negative	Healthy	Epidemic	5	A	
379	22.05.2020	26,87	40,18	55,61	223,97	1159,24	182,41	60	Female	Health Service	Negative	Negative	Positive	Negative	Positive	Negative	Healthy	Epidemic	3	A	
380	22.05.2020	23,36	36,71	2,98	282,91	327,93	220,89	44	Female	Health Service	Negative	Negative	Negative	Negative	Negative	Negative	Healthy	Epidemic	0	A	
381	22.05.2020	24,53	41,67	62,07	92,83	703,29	126,94	32	Female	Health Service	Negative	Negative	Positive	Negative	Positive	Negative	Healthy	Epidemic	4	A	
382	22.05.2020	22,20	34,23	9,43	183,20	439,98	148,43	53	Female	Health Service	Negative	Positive	Positive	Negative	Negative	Positive	Healthy	Epidemic	5	A	
383	22.05.2020	24,53	61,51	1,49	180,26	337,85	255,37	No_Data	Female	Health Service	Negative	Negative	Negative	Negative	Negative	Negative	Healthy	Epidemic	0	A	
384	22.05.2020	14,02	22,32	2,48	69,74	183,45	99,95	49	Female	Health Service	Negative	Negative	Negative	Negative	Negative	Negative	Healthy	Epidemic	0	A	
385	22.05.2020	21,03	39,19	9,93	215,62	844,39	209,90	45	Female	Health Service	Negative	Negative	Negative	Negative	Negative	Negative	Healthy	Epidemic	1	A	
386	22.05.2020	35,83	53,32	281,78	163,06		226,14	50	Female	Health Service	Negative	Negative	Negative	Negative	Negative	Negative	Healthy	Epidemic	0	A	
387	22.05.2020	18,69	23,31	1,99	83,50	272,75	107,45	50	Male	Office	Positive	Positive	Positive	Negative	Positive	Negative	Healthy	Epidemic	3	A	
388	22.05.2020	18,69	29,27	1,49	101,18	401,50	169,92	42	Female	Health Service	Negative	Negative	Positive	Negative	Positive	Negative	Healthy	Epidemic	2	A	
389	22.05.2020	17,52	41,67	6,95	114,93	214,18	325,84	26	Male	Health Service	Negative	Negative	Negative	Negative	Negative	Negative	Healthy	Epidemic	0	A	
390	22.05.2020	23,36	48,61	22,84	82,51	185,87	113,94	55	Female	Health Service	Negative	Positive	Positive	Negative	Positive	Positive	Healthy	Epidemic	6	A	
391	22.05.2020	18,69	37,20	13,90	166,50	699,18	144,43	66	Female	Health Service	Negative	Negative	Negative	Negative	Negative	Negative	Healthy	Epidemic	0	A	
392	22.05.2020	25,70	25,79	7,45	84,97	912,63	71,96	49	Female	Health Service	Negative	Positive	Positive	Negative	Positive	Negative	Healthy	Epidemic	5	A	
393	22.05.2020	21,03	375,00	2,48	396,86	645,45	659,17	48	Male	Health Service	Negative	Negative	Positive	Negative	Positive	Positive	Healthy	Epidemic	4	A	
394	22.05.2020	23,36	23,31	5,96	181,24	398,35	170,91	27	Female	Health Service	Negative	Negative	Negative	Negative	Negative	Negative	Healthy	Epidemic	1	A	
395	24.05.2020	58,41	29,27	638,78	175,34		140,43	21	Female	Student	Negative	Negative	Negative	Negative	Negative	Negative	Healthy	Epidemic	1	A	
395B	12.10.2020	37,38	23,81	371,40	58,94	515,00	88,96	21	Female	Student	Negative	Negative	Negative	Negative	Negative	Negative	Healthy	Epidemic	1	B	
396	24.05.2020	168,22	248,51	450,10	228,39	539,69	361,32	46	Male	Education	Negative	Positive	Negative	Negative	Negative	Negative	Healthy	Epidemic	3	A	
396B	08.10.2020	37,38	56,55	393,25	123,77	723,14	132,93	46	Male	Education	Negative	Positive	Negative	Negative	Negative	Negative	Healthy	Epidemic	3	B	
397	06.07.2020	16,36	21,83	139,52	53,54	213,21	52,47	25	Female	Health Service	Negative	Positive	Negative	Negative	Positive	Negative	Healthy	Epidemic	4	A	
398	28.05.2020	16,36	77,88	107,25	173,38	171,35	141,43	46	Female	Health Service	Negative	Positive	Positive	Negative	Negative	Negative	Healthy	Epidemic	3	A	
399	03.06.2020	44,39	57,54	768,62	1404,72	1197,48		No_Data	No_Data	No_Data	No_Data	No_Data	No_Data	No_Data	No_Data	No_Data	Healthy	Epidemic	0	A	
400	28.05.2020	30,37	67,46	3,48	188,85	114,71	234,38	55	Female	Health Service	Negative	Negative	Negative	Negative	Negative	Negative	Healthy	Epidemic	0	A	
401	23.05.2020	14,02	55,06	3,97	211,20		261,87	42	Female	Health Service	Negative	Negative	Negative	Negative	Negative	Negative	Healthy	Epidemic	0	A	
402	23.05.2020	11,68	37,20	3,48	114,93	1,94	188,91	49	Female	Health Service	Negative	Negative	Negative	Negative	Positive	Negative	Healthy	Epidemic	1	A	
403	23.05.2020	28,04	129,96	170,80	315,32	2,90	362,32	60	Female	No_Data	Negative	Negative	Positive	Negative	Positive	Negative	Healthy	Epidemic	2	A	
404	23.05.2020	18,69	39,68	126,61	98,72	4,36	91,45	46	Male	Health Service	Negative	Negative	Negative	Negative	Negative	Negative	Healthy	Epidemic	0	A	
**COVID**																					
Sample	Sample Date	Anty-N IgG	Anty-N IgA	Anty-S1 IgG	Anty-S1 IgA	Anty-E IgG	Anty-E IgA	Age	Gender	Workplace	Temperature Over 38	Temperature over 37	Cough	Breath difficulties	Runny nose	Smell and taste disorders	Status	Date Status	Number of Symptoms	Sample order	Course of illness (COVID only)
COV1	26.04.2020	37,85	129,96	630,71	1004,67	1024,08	877,56	69	Female	No_Data	Negative	Negative	Negative	Negative	Negative	Negative	COVID	Epidemic	0	A	
COV10	05.05.2020	22,20	61,26	174,78	478,39	641,34	718,64	No_Data	No_Data	No_Data	No_Data	No_Data	No_Data	No_Data	No_Data	No_Data	COVID	Epidemic	0	A	No_data
COV11	05.05.2020	827,10	543,15	1182,72	703,34	504,84	633,18	47	Male	No_Data	Positive	Positive	Positive	Negative	Negative	Negative	COVID	Epidemic	3	A	severe
COV12	05.05.2020	14,02	17,61	42,20	86,94	232,33	251,37	31	Female	No_Data	Negative	Negative	Negative	Positive	Negative	Negative	COVID	Epidemic	2	A	mild
COV13	05.05.2020	144,86	187,25	92,85	89,64	900,94	229,89	53	Female	Health Service	Negative	Negative	Negative	Negative	Negative	Negative	COVID	Epidemic	1	A	mild
COV14	05.05.2020	24,53	25,05	337,64	70,24	515,97	186,91	24	Female	No_Data	Negative	Negative	Negative	Negative	Negative	Negative	COVID	Epidemic	0	A	asymptomatic
COV15	05.05.2020	543,22	1181,55	229,89	218,57	1091,80	703,15	58	Female	Health Service	Negative	Positive	Positive	Negative	Negative	Negative	COVID	Epidemic	3	A	mild
COV16	05.05.2020	1000,00	1000,00	1000,00	1000,00	1000,00	1000,00	71	Female	No_Data	Negative	Negative	Negative	Negative	Negative	Negative	COVID	Epidemic	0	A	asymptomatic
COV17	05.05.2020	742,99		703,08	195,73	1517,91	178,91	44	Female	No_Data	Positive	Positive	Positive	Positive	Negative	Negative	COVID	Epidemic	5	A	critical
COV18	05.05.2020	261,68	105,65	609,24	389,49	604,07	507,75	No_Data	No_Data	No_Data	Positive	Positive	Positive	Positive	Negative	Positive	COVID	Epidemic	7	A	severe
COV19	15.05.2020	37,38	85,81	847,57	769,16	249,76	994,00	50	Female	Health Service	Negative	Negative	Negative	Negative	Negative	Negative	COVID	Epidemic	0	A	asymptomatic
COV2	05.05.2020	262,46	59,52	397,47	317,78	1095,84	1031,48	70	Male	No_Data	Positive	Positive	Positive	Positive	Negative	Negative	COVID	Epidemic	3	A	moderate
COV20	19.05.2020	44,39	237,10	452,83	317,29	227,01	406,80	46	Male	No_Data	Negative	Positive	Negative	Negative	Negative	Negative	COVID	Epidemic	4	A	moderate
COV21	19.05.2020	39,72	33,23	105,76	101,18	136,98	58,97	23	Female	Office	Negative	Positive	Negative	Negative	Positive	Positive	COVID	Epidemic	4	A	moderate
COV21B	14.10.2020	18,69	13,89	127,11	39,29	156,82	29,99	23	Female	Office	Negative	Positive	Negative	Negative	Positive	Positive	COVID	Epidemic	4	B	moderate
COV22A	19.05.2020	607,48	94,25	431,48	274,56	242,26	185,66	55	Female	Education	Positive	Positive	Positive	Negative	Positive	Negative	COVID	Epidemic	6	A	severe
COV22B	14.10.2020	102,80	24,80	307,85	128,68	355,28	125,94	55	Female	Education	Positive	Positive	Positive	Negative	Positive	Negative	COVID	Epidemic	6	B	severe
COV23A	19.05.2020	63,08	59,52	97,32	148,33	433,69	107,70	57	Male	Office	Negative	Positive	Negative	Negative	Negative	Negative	COVID	Epidemic	3	A	moderate
COV23B	14.10.2020	28,04	27,78	187,69	83,50	294,29	52,97	57	Male	Office	Negative	Positive	Negative	Negative	Negative	Negative	COVID	Epidemic	3	B	moderate
COV24A	29.05.2020	1028,04	93,01	525,49	94,06	1215,88	109,95	47	Female	Office	Negative	Negative	Positive	Positive	Positive	Positive	COVID	Epidemic	5	A	severe
COV24B	12.10.2020	186,92	32,74	424,03	58,94	987,42	52,97	47	Female	Office	Negative	Negative	Positive	Positive	Positive	Positive	COVID	Epidemic	5	B	severe
COV25A	12.06.2020	108,64	47,37	728,90	182,47	750,24	83,21	48	Female	Health Service	Positive	Positive	Positive	Positive	Positive	Positive	COVID	Epidemic	8	A	severe
COV25B	12.10.2020	56,07	24,80	729,89	128,68	723,14	41,98	48	Female	Health Service	Positive	Positive	Positive	Positive	Positive	Positive	COVID	Epidemic	8	B	severe

COV26B	04.06.2020	82,94	177,83	835,15	673,87	770,45	146,09	51	Female	Health Service	Negative	Negative	Positive	Negative	Negative	Positive	COVID	Epidemic	3	B	mild
COV26C	09.06.2020	98,13	103,67	1305,86	565,32	388,19	116,44	51	Female	Health Service	Negative	Negative	Positive	Negative	Negative	Positive	COVID	Epidemic	3	C	mild
COV26D	24.09.2020	28,04	34,72	516,39	120,83	766,70	64,97	51	Female	Health Service	Negative	Negative	Positive	Negative	Negative	Positive	COVID	Epidemic	3	D	mild
COV27	09.06.2020	75,93	31,00	1932,47	100,93	1692,88	84,46	35	Male	No_Data	Negative	Negative	Negative	Negative	Negative	Negative	COVID	Epidemic	0	A	asymptomatic
COV28	09.06.2020	44,39	55,06	332,67	141,94	315,59	186,66	28	Male	Health Service	Negative	Negative	Negative	Negative	Negative	Negative	COVID	Epidemic	0	A	asymptomatic
COV28B	21.07.2020	28,04	37,70	215,49	83,50	220,72	55,97	28	Male	Health Service	Negative	Negative	Negative	Negative	Negative	Negative	COVID	Epidemic	0	B	asymptomatic
COV28C	24.09.2020	28,04	31,75	210,53	77,60	173,28	76,96	28	Male	Health Service	Negative	Negative	Negative	Negative	Negative	Negative	COVID	Epidemic	0	C	asymptomatic
COV29A	09.06.2020	91,12	70,93	462,76	139,00	546,47	148,43	29	Male	Health Service	Negative	Negative	Negative	Negative	Negative	Negative	COVID	Epidemic	0	A	asymptomatic
COV29B	21.07.2020	93,46	55,56	349,55	119,84	223,86	53,47	29	Male	Health Service	Negative	Negative	Negative	Negative	Negative	Negative	COVID	Epidemic	0	B	asymptomatic
COV29C	24.09.2020	116,82	39,68	307,85	99,21	159,73	65,97	29	Male	Health Service	Negative	Negative	Negative	Negative	Negative	Negative	COVID	Epidemic	0	C	asymptomatic
COV3	05.05.2020	729,75	300,60	393,99	291,75	191,51	440,78	30	Male	No_Data	Negative	Negative	Negative	Negative	Negative	Negative	COVID	Epidemic	0	A	asymptomatic
COV30A	09.06.2020	299,07			449,90	808,33	919,79	85	Male	No_Data	Negative	Negative	Negative	Negative	Negative	Negative	COVID	Epidemic	0	A	asymptomatic
COV4	05.05.2020	53,15	40,34	386,05	203,59	389,16	270,36	24	Female	Health Service	Negative	Negative	Negative	Negative	Negative	Negative	COVID	Epidemic	0	A	asymptomatic
COV5	05.05.2020	53,27	57,66	302,71	138,26	255,24	155,42	56	Male	No_Data	Positive	Positive	Positive	Positive	Negative	Negative	COVID	Epidemic	4	A	moderate
COV6	05.05.2020	172,31	103,84	115,52	131,63	514,36	197,23	49	Female	Health Service	Negative	Negative	Positive	Negative	Negative	Negative	COVID	Epidemic	2	A	mild
COV7	05.05.2020	32,71	25,63	38,73	148,98	1136,50	96,12	78	Female	No_Data	Negative	Negative	Negative	Negative	Negative	Negative	COVID	Epidemic	0	A	asymptomatic
COV8	05.05.2020	41,59	75,52	64,30	120,46	297,80	186,74	49	Female	Health Service	Negative	Positive	Positive	Negative	Negative	Negative	COVID	Epidemic	2	A	mild
COV9	05.05.2020	78,27	18,11	31,78	69,25	184,41	55,47	30	Male	No_Data	Negative	Negative	Negative	Negative	Negative	Negative	COVID	Epidemic	0	A	asymptomatic
COV19B	27.11.2020	57,41627	72	354,5	285	571,8623		50	Female	Health Service	Negative	Negative	Negative	Negative	Negative	Negative	COVID	Epidemic	0	B	asymptomatic
COV19C	05.12.2020	52,63158	78	309,5	120,5	444,332		50	Female	Health Service	Negative	Negative	Negative	Negative	Negative	Negative	COVID	Epidemic	0	C	asymptomatic
COV20B	27.11.2020	33,49282	132	198,5	59	211,5		46	Male	Army	Negative	Positive	Negative	Negative	Negative	Negative	COVID	Epidemic	4	B	moderate
COV20C	05.12.2020	94,09888	116	400,5	98,5	825,9109		46	Male	Army	Negative	Positive	Negative	Negative	Negative	Negative	COVID	Epidemic	4	C	moderate

Historical

Sample	Sample Date	Anty-N IgG	Anty-N IgA	Anty-S1 IgG	Anty-S1 IgA	Anty-E IgG	Anty-E IgA	Age	Gender	Workplace	Temperature Over 38	Temperature over 37	Cough	Breath difficulties	Runny nose	Smell and taste disorders	Status	Date Status	Number of Symptoms	Sample order	Course of illness (COVID only)
HIS1	2010	39,72	20,34	234,86	82,51	525,65	107,95	24	Female	No_Data	No_Data	No_Data	No_Data	No_Data	No_Data	No_Data	Historical	<2019		A	
HIS10	2010	37,38	14,38	180,73	249,51	288,00	236,38	31	Male	No_Data	No_Data	No_Data	No_Data	No_Data	No_Data	No_Data	Historical	<2019		A	
HIS11	2010	18,69	16,87	230,39	147,84	179,09	135,43	28	Male	No_Data	No_Data	No_Data	No_Data	No_Data	No_Data	No_Data	Historical	<2019		A	
HIS12	2010	11,68	11,41	1314,80	80,55	442,40	185,41	25	Male	No_Data	No_Data	No_Data	No_Data	No_Data	No_Data	No_Data	Historical	<2019		A	
HIS13	2010	228,97	15,87	449,35	322,20	553,73	226,39	29	Female	No_Data	No_Data	No_Data	No_Data	No_Data	No_Data	No_Data	Historical	<2019		A	
HIS14	2010	51,40	9,92	1074,48	71,71		67,97	22	Female	No_Data	No_Data	No_Data	No_Data	No_Data	No_Data	No_Data	Historical	<2019		A	
HIS15	2010	35,05	16,87	569,02	217,09	248,79	167,92	25	Male	No_Data	No_Data	No_Data	No_Data	No_Data	No_Data	No_Data	Historical	<2019		A	
HIS16	2010	56,07	40,67	682,72	302,06	657,79	450,77	25	Male	No_Data	No_Data	No_Data	No_Data	No_Data	No_Data	No_Data	Historical	<2019		A	
HIS17	2010	67,76	27,78	1017,87	156,68	468,05	149,93	27	Female	No_Data	No_Data	No_Data	No_Data	No_Data	No_Data	No_Data	Historical	<2019		A	
HIS18	2010	46,73	118,55	668,82	835,46	1336,88	835,58	33	Male	No_Data	No_Data	No_Data	No_Data	No_Data	No_Data	No_Data	Historical	<2019		A	
HIS19	2010	51,40	59,03	3,97	243,12		264,37	24	Male	No_Data	No_Data	No_Data	No_Data	No_Data	No_Data	No_Data	Historical	<2019		A	
HIS2	2010	14,02	12,40	408,14	58,94	1178,61	44,98	27	Female	No_Data	No_Data	No_Data	No_Data	No_Data	No_Data	No_Data	Historical	<2019		A	
HIS20	2010	37,38	32,74	538,73	303,54	307,84	133,43	25	Male	No_Data	No_Data	No_Data	No_Data	No_Data	No_Data	No_Data	Historical	<2019		A	
HIS21	2010	37,38	17,36	336,15	291,26	431,75	153,42	21	Female	No_Data	No_Data	No_Data	No_Data	No_Data	No_Data	No_Data	Historical	<2019		A	
HIS22	2010	25,70	22,32	415,09	152,26	455,95	83,46	22	Male	No_Data	No_Data	No_Data	No_Data	No_Data	No_Data	No_Data	Historical	<2019		A	
HIS23	2010	25,70	35,71	224,93	206,78	393,51	205,40	32	Male	No_Data	No_Data	No_Data	No_Data	No_Data	No_Data	No_Data	Historical	<2019		A	
HIS24	2010	39,72	35,22	579,44	256,39	636,01	164,42	19	Male	No_Data	No_Data	No_Data	No_Data	No_Data	No_Data	No_Data	Historical	<2019		A	
HIS25	2010	46,73	19,84	487,09	145,87	443,37	278,86	21	Male	No_Data	No_Data	No_Data	No_Data	No_Data	No_Data	No_Data	Historical	<2019		A	
HIS3	2010	46,73	62,50	1003,97	257,37	938,04	291,85	27	Female	No_Data	No_Data	No_Data	No_Data	No_Data	No_Data	No_Data	Historical	<2019		A	
HIS4	2010	30,37	54,56	505,96	343,81	328,17	269,87	25	Male	No_Data	No_Data	No_Data	No_Data	No_Data	No_Data	No_Data	Historical	<2019		A	
HIS5	2010	44,39	58,04	548,66	238,21	588,58	296,35	27	Male	No_Data	No_Data	No_Data	No_Data	No_Data	No_Data	No_Data	Historical	<2019		A	
HIS6	2010	58,41	34,23	508,44	273,58	466,60	197,40	25	Male	No_Data	No_Data	No_Data	No_Data	No_Data	No_Data	No_Data	Historical	<2019		A	
HIS7	2010	35,05	35,71	482,62	258,84	939,01	169,92	24	Male	No_Data	No_Data	No_Data	No_Data	No_Data	No_Data	No_Data	Historical	<2019		A	
HIS8	2010	49,07	37,20	531,78	76,62	441,43	95,45	25	Male	No_Data	No_Data	No_Data	No_Data	No_Data	No_Data	No_Data	Historical	<2019		A	
HIS9	2010	35,05	39,19	321,25	235,76	827,69	313,84	25	Male	No_Data	No_Data	No_Data	No_Data	No_Data	No_Data	No_Data	Historical	<2019		A	
242 in 2017	2017	53,74	90,77	447,86	368,37	1057,60	219,89	27	Female	No_Data	No_Data	No_Data	No_Data	No_Data	No_Data	No_Data	Historical	<2019		A	
244 in 2017	2017	14,02	16,87	546,18	72,69	436,11	244,88	26	Male	No_Data	No_Data	No_Data	No_Data	No_Data	No_Data	No_Data	Historical	<2019		A	
HIS113	2017	11,68	45,14	110,72	156,68	185,87	238,88	No_Data	No_Data	No_Data	No_Data	No_Data	No_Data	No_Data	No_Data	No_Data	Historical	<2019		A	
HIS114	2017	42,06	19,35	743,79	115,91	77,44	44,48	No_Data	No_Data	No_Data	No_Data	No_Data	No_Data	No_Data	No_Data	No_Data	Historical	<2019		A	
HIS116	2017	4,67	65,48	72,00	231,83	241,53	191,90	No_Data	No_Data	No_Data	No_Data	No_Data	No_Data	No_Data	No_Data	No_Data	Historical	<2019		A	
HIS117	2017	21,03	39,19	101,79	134,58	105,52	92,45	No_Data	No_Data	No_Data	No_Data	No_Data	No_Data	No_Data	No_Data	No_Data	Historical	<2019		A	
HIS118	2017	25,70	33,23	287,49	98,23	104,07	76,46	No_Data	No_Data	No_Data	No_Data	No_Data	No_Data	No_Data	No_Data	No_Data	Historical	<2019		A	
HIS119	2017	32,71	17,36	869,91	87,92	206,20	66,47	No_Data	No_Data	No_Data	No_Data	No_Data	No_Data	No_Data	No_Data	No_Data	Historical	<2019		A	
HIS120	2017	25,70	85,32	286,00	526,03	157,79	238,38	No_Data	No_Data	No_Data	No_Data	No_Data	No_Data	No_Data	No_Data	No_Data	Historical	<2019		A	
HIS121	2017	16,36	90,77	270,61	217,58	293,32	737,13	No_Data	No_Data	No_Data	No_Data	No_Data	No_Data	No_Data	No_Data	No_Data	Historical	<2019		A	
HIS122	2017	28,04	65,48	607,75	232,81	233,79	235,38	No_Data	No_Data	No_Data	No_Data	No_Data	No_Data	No_Data	No_Data	No_Data	Historical	<2019		A	
HIS123	2017	9,35	12,90	125,62	78,59	281,22	22,49	No_Data	No_Data	No_Data	No_Data	No_Data	No_Data	No_Data	No_Data	No_Data	Historical	<2019		A	
HIS125	2017	11,68	18,85	82,92	69,25	343,66	27,99	No_Data	No_Data	No_Data	No_Data	No_Data	No_Data	No_Data	No_Data	No_Data	Historical	<2019		A	
HIS126	2017	30,37	19,84	476,17	158,15	347,05	74,46	No_Data	No_Data	No_Data	No_Data	No_Data	No_Data	No_Data	No_Data	No_Data	Historical	<2019		A	
HIS127	2017	23,36	16,87	171,30	85,95	1134,56	53,97	No_Data	No_Data	No_Data	No_Data	No_Data	No_Data	No_Data	No_Data	No_Data	Historical	<2019		A	
HIS128	2017	56,07	28,27	463,26	325,64	256,05	101,95	No_Data	No_Data	No_Data	No_Data	No_Data	No_Data	No_Data	No_Data	No_Data	Historical	<2019		A	
HIS129	2017	9,35	19,84	32,77	131,63	319,94	72,96	No_Data	No_Data	No_Data	No_Data	No_Data	No_Data	No_Data	No_Data	No_Data	Historical	<2019		A	
HIS130	2017	32,71	29,27	145,98	108,55	302,52	63,97	No_Data	No_Data	No_Data	No_Data	No_Data	No_Data	No_Data	No_Data	No_Data	Historical	<2019		A	
HIS131	2017	28,04	38,19	231,38	109,53	232,82	159,92	No_Data	No_Data	No_Data	No_Data	No_Data	No_Data	No_Data	No_Data	No_Data	Historical	<2019		A	
HIS132	2017	32,71	57,54	599,80	479,86	153,92	143,93	No_Data	No_Data	No_Data	No_Data	No_Data	No_Data	No_Data	No_Data	No_Data	Historical	<2019		A	
HIS133	2017	14,02	21,33	122,14	81,04	179,57	93,95	No_Data	No_Data	No_Data	No_Data	No_Data	No_Data	No_Data	No_Data	No_Data	Historical	<2019		A	
HIS170	2017	25,70	26,29	186,20	144,40	208,13	124,94	57	Male	No_Data	No_Data	No_Data	No_Data	No_Data	No_Data	No_Data	Historical	<2019		A	
HIS171	2017	44,39	43,15	701,09	222,99	328,17	110,94	31	Female	No_Data	No_Data	No_Data	No_Data	No_Data	No_Data	No_Data	Historical	<2019		A	
HIS172	2017	32,71	30,26	399,70	69,74	173,28	31,98	35	Female	No_Data	No_Data	No_Data	No_Data	No_Data	No_Data	No_Data	Historical	<2019		A	
HIS173	2017	49,07	40,18	968,22	155,70	585,19	95,95	22	Male	No_Data	No_Data	No_Data	No_Data	No_Data	No_Data	No_Data	Historical	<2019		A	
HIS174	2017	18,69	18,35	190,17	63,85	187,80	60,47	25	Female	No_Data	No_Data	No_Data	No_Data	No_Data	No_Data	No_Data	Historical	<2019		A	
HIS175	2017	18,69	13,39	284,01	68,27	1085,67	41,48	23	Female	No_Data	No_Data	No_Data	No_Data	No_Data	No_Data	No_Data	Historical	<2019		A	
HIS176	2017	14,02	15,87	70,51	31,43	132,14	18,99	27	Female	No_Data	No_Data	No_Data	No_Data	No_Data	No_Data	No_Data	Historical	<2019		A	
HIS177	2017	21,03	53,08	174,78	94,30	235,72	101,95	26	Female	No_Data	No_Data	No_Data	No_Data	No_Data	No_Data	No_Data	Historical	<2019		A	
HIS178	2017	7,01		120,16		228,94	2,50	20	Male	No_Data	No_Data	No_Data	No_Data	No_Data	No_Data	No_Data	Historical	<2019		A	
HIS179	2017	14,02	86,81	81,93	13,75	359,63	262,37	23	Male	No_Data	No_Data	No_Data	No_Data	No_Data	No_Data	No_Data	Historical	<2019		A	
HIS180	2017	16,36	46,63	134,56	221,02	247,34	113,44	21	Female	No_Data	No_Data	No_Data	No_Data	No_Data	No_Data	No_Data	Historical	<2019		A	
HIS181	2017	37,38	73,41	140,52	132,61	317,52	166,92	21	Female	No_Data	No_Data	No_Data	No_Data	No_Data	No_Data	No_Data	Historical	<2019		A	
HIS182	2017	16,36	18,85	50,65	62,38	277,35	47,98	26	Male	No_Data	No_Data	No_Data	No_Data	No_Data	No_Data	No_Data	Historical	<2019		A	
HIS183	2017	14,02	22,32		69,74	827,69	1246,38	28	Male	No_Data	No_Data	No_Data	No_Data	No_Data	No_Data	No_Data	Historical	<2019		A	
HIS184	2017	18,69	32,74		197,45	480,64	152,42	40	Male	No_Data	No_Data	No_Data	No_Data	No_Data	No_Data	No_Data	Historical	<2019		A	
HIS185	2017	25,70	38,69		415,52	138,92	55,97	31	Female	No_Data	No_Data	No_Data	No_Data	No_Data	No_Data	No_Data	Historical	<2019		A	

HIS186	2017	11,68	8,93		88,90	220,72	89,96	28	Male	No_Data	No_Data	No_Data	No_Data	No_Data	No_Data	No_Data	Historical	<2019		A	
HIS187	2017	7,01	4,96		25,54	244,92	523,24	31	Female	No_Data	No_Data	No_Data	No_Data	No_Data	No_Data	No_Data	Historical	<2019		A	
E181	10.12.2019	51,40	45,14	248,26	155,70	786,87	186,66	71	Male	No_Data	No_Data	No_Data	No_Data	No_Data	No_Data	No_Data	Historical	Pre-epidemic		A	
E182	10.12.2019	46,73	49,60	548,16	157,17	615,68	187,91	58	Male	No_Data	No_Data	No_Data	No_Data	No_Data	No_Data	No_Data	Historical	Pre-epidemic		A	
E196	28.01.2020	39,72	98,21		196,46	527,83	490,75	72	Female	No_Data	No_Data	No_Data	No_Data	No_Data	No_Data	No_Data	Historical	Pre-epidemic		A	
E198	28.01.2020	42,06	24,80	19,86	165,52	1112,78	108,95	60	Female	No_Data	No_Data	No_Data	No_Data	No_Data	No_Data	No_Data	Historical	Pre-epidemic		A	
E201	28.01.2020	49,65	75,64	23,50	264,98	1121,97	565,97	73	Male	No_Data	No_Data	No_Data	No_Data	No_Data	No_Data	No_Data	Historical	Pre-epidemic		A	
E222	03.03.2020	33,49	132,69	442,40	445,48	1602,13	752,37	52	Male	No_Data	No_Data	No_Data	No_Data	No_Data	No_Data	No_Data	Historical	Pre-epidemic		A	
E226	10.03.2020	92,68	101,19	523,01	428,05	1959,34	645,43	50	Male	No_Data	No_Data	No_Data	No_Data	No_Data	No_Data	No_Data	Historical	Pre-epidemic		A	
E227	10.03.2020	32,71	29,76	4,47	139,98	968,05	249,88	64	Male	No_Data	No_Data	No_Data	No_Data	No_Data	No_Data	No_Data	Historical	Pre-epidemic		A	

Table S2. Statistics for serum levels of specific antibodies targeting SARS-CoV-2 structural proteins N, S (S1 domain), and E, in patients with confirmed SARS-CoV-2 infection (COVID), Healthy Volunteers – blood donors without confirmed SARS-CoV-2 infection (Healthy), serum samples collected from healthy volunteers in the years 2010-2017 (Historical). Presented values are normalized ELISA units, and column 'Age' gives the values in years; N – number of samples analyzed in the group (according to data availability), Min – the lowest value in the group, Max – the highest value in the group, 1stQu – the first quartile cutoff, 3rdQu – the third quartile cutoff, Median – median value in the group, Mean – mean value in the group.

		Anti-N IgG	Anti-N IgA	Anti-S1 IgG	Anti-S1 IgA	Anti-E IgG	Anti-E IgA	Age
**COVID**	Min	14.02	17.61	31.78	69.25	137	55.47	23
	1stQu	41.59	43.86	113.08	120.46	255.2	148.43	33
	Median	78.27	70.93	390.02	182.47	516	197.23	48
	Mean	257.97	175.56	451.82	288.88	645.4	379.54	47.89
	3rd Qu	299.07	117.81	614.6	317.78	1000	633.18	56.5
	Max	1028.04	1181.55	1932.47	1004.67	1692.9	1031.48	85
	N	29	27	28	29	29	29	27
**Healthy**	Min	7.009	5.704	1.49	36.35	1.936	21.99	19
	1stQu	18.692	25.422	84.91	105.6	182.72	112.69	33
	Median	24.533	42.659	186.69	152.75	347.209	189.91	43
	Mean	28.111	57.992	253.51	197.2	417.202	255.33	42.88
	3rd Qu	31.25	63.74	349.8	223.35	590.513	313.84	51
	Max	168.224	667.411	1098.31	1404.72	1417.231	1402.8	76
	N	226	226	225	225	209	223	206
**Historical**	Min	4.673	4.96	3.972	13.75	77.44	2.499	19
	1stQu	16.355	17.86	158.639	81.78	229.91	74.088	24
	Median	28.037	30.26	336.147	152.26	318.73	139.68	25
	Mean	32.601	35.79	400.291	186.61	419.47	187.133	26.8
	3rd Qu	40.304	44.15	547.418	246.32	477.49	235.632	28
	Max	228.972	118.55	1314.796	835.46	1336.88	1246.377	57
	N	64	63	61	63	62	64	45

Table S3. Inhibition of binding of ACE2 receptor and virus protein S, cell-free competitive ELISA kit. Sera were tested with SARS-CoV-2 Neutralizing Antibody Detection ELISA Kit. Sera were selected according to identified levels of SARS-CoV-2 protein-specific IgG specified in the column 'IgG antibodies status' (for details see Supplementary material: Table S1): + inhibition observed, – inhibition not observed, ? result inconclusive, positive reference – all types of IgG high and virus neutralization confirmed in cultures, negative reference – all types of IgG low and virus neutralization in cultures not observed.

	Inhibition of S protein-ACE2 receptor binding	SARS-CoV-2 infection	comments
COV15	+	**Infection confirmed**	positive reference
COV11	+	**Infection confirmed**	positive reference
COV18	+	**Infection confirmed**	positive reference
HIS178	-	Historical collection of sera (2017)	negative reference
371	-	Healthy/undiagnosed	negative reference
COV19 A	?	**Infection confirmed**	
COV19 B	+	**Infection confirmed**	Secondly infected
COV20 A	?	**Infection confirmed**	
COV20 B	+	**Infection confirmed**	Secondly infected
COV21 A	+	**Infection confirmed**	
COV 21 B	+	**Infection confirmed**	
COV22 A	+	**Infection confirmed**	
COV22 B	+	**Infection confirmed**	
COV23 A	+	**Infection confirmed**	
COV23 B	+	**Infection confirmed**	
COV24 A	+	**Infection confirmed**	
COV24 B	+	**Infection confirmed**	
COV25 A	+	**Infection confirmed**	
COV25 B	+	**Infection confirmed**	
COV26 B	+	**Infection confirmed**	
COV26 C	+	**Infection confirmed**	
COV26 D	+	**Infection confirmed**	
COV28 A	+	**Infection confirmed**	
COV28 B	+	**Infection confirmed**	
COV28 C	+	**Infection confirmed**	
COV29 A	+	**Infection confirmed**	
COV29 B	+	**Infection confirmed**	
COV29 C	+	**Infection confirmed**	
395 A	?	Healthy/undiagnosed	Undiagnosed infection highly probable
395 B	?	Healthy/undiagnosed	Undiagnosed infection highly probable
396 B	+	Healthy/undiagnosed	Undiagnosed infection highly probable
396 B	+	Healthy/undiagnosed	Undiagnosed infection highly probable
248A	?	Healthy/undiagnosed	Undiagnosed infection highly probable
248C	?	Healthy/undiagnosed	Undiagnosed infection highly probable

HIS3	-	Historical collection	
HIS8	-	Historical collection	
HIS12	-	Historical collection	
HIS13	-	Historical collection	
HIS14	-	Historical collection	
HIS16	-	Historical collection	
HIS17	-	Historical collection	
HIS114	-	Historical collection	
HIS119	-	Historical collection	
HIS171	-	Historical collection	
HIS175	-	Historical collection	
HIS178	-	Historical collection	
E182	-	Pre-epidemic	
E198	-	Pre-epidemic	
E226	-	Pre-epidemic	
